# Single Nucleotide Mutagenesis of the *TaCHLI* Gene Suppressed Chlorophyll and Fatty Acid Biosynthesis in Common Wheat Seedlings

**DOI:** 10.3389/fpls.2020.00097

**Published:** 2020-02-20

**Authors:** Chaojie Wang, Lili Zhang, Yingzhuang Li, Zeeshan Ali Buttar, Na Wang, Yanzhou Xie, Chengshe Wang

**Affiliations:** ^1^College of Agronomy, Northwest A&F University, Yangling, China; ^2^State Key Laboratory of Crop Stress Biology for Arid Areas, Northwest A&F University, Yangling, China

**Keywords:** common wheat, pale-green mutant, gene clone, *CHLI*, protoporphyrin IX, lipid

## Abstract

Wheat (*Triticum aestivum* L.) is one of the most important crops in the world. Chlorophyll plays a vital role in plant development and crop improvement and further determines the crop productivity to a certain extent. The biosynthesis of chlorophyll remains a complex metabolic process, and fundamental biochemical discoveries have resulted from studies of plant mutants with altered leaf color. In this study, we identified a chlorophyll-deficiency mutant, referred to as *chli*, from the wheat cultivar Shaannong33 that exhibited an obvious pale-green leaf phenotype at the seedling stage, with significantly decreased accumulation of chlorophyll and its precursors, protoporphyrin IX and Mg-protoporphyrin IX. Interestingly, a higher protoporphyrin IX to Mg-protoporphyrin IX ratio was observed in *chli*. Lipid biosynthesis in *chli* leaves and seeds was also affected, with the mutant displaying significantly reduced total lipid content relative to Shaanong33. Genetic analysis indicated that the pale-green leaf phenotype was controlled by a single pair of recessive nuclear genes. Furthermore, sequence alignment revealed a single-nucleotide mutation (G664A) in the gene TraesCS7A01G480700.1, which encodes subunit I of the Mg-chelatase in plants. This single-nucleotide mutation resulted in an amino acid substitution (D221N) in the highly conserved domain of subunit I. As a result, mutant protein Tachli-7A lost the ability to interact with the normal protein TaCHLI-7A, as assessed by yeast two-hybrid assay. Meanwhile, *Tachli-7A* could not recover the chlorophyll deficiency phenotype of the *Arabidopsis thaliana* SALK_050029 mutant. Furthermore, we found that in Shaannong33, the protoporphyrin IX to Mg-protoporphyrin IX ratio was growth state-dependent and insensitive to environmental change. Overall, the mutation in Tachli-7A impaired the function of Mg-chelatase and blocked the conversion of protoporphyrin IX to Mg- protoporphyrin IX. Based on our results, the *chli* mutant represents a potentially useful resource for better understanding chlorophyll and lipid biosynthetic pathways in common wheat.

## Introduction

Wheat (*Triticum aestivum* L.) is one of the most important crops in the world. Nearly 35% of the human population obtains substantial caloric intake from wheat-derived food (Paux et al., 2008). As such, enhancing wheat yield remains a major goal of crop breeding (Emamgholizadeh et al., 2015). Photosynthesis plays a critical role in crop productivity and is therefore a target for plant improvement (Nasyrov, 1978). During photosynthesis, chlorophyll (Chl) pigments play key roles in light absorption (Fromme et al., 2003). It is generally believed that higher levels of solar energy conversion to chemical energy may result in a certain degree of enhanced crop biomass accumulation (Chen and Blankenship, 2011). During photosynthesis, Chl absorbs light energy, representing the first step in the conversion of light energy into chemical D:\Application Data\Microsoft\Local Settings\Application Data\youdao\dict\Application\7.5.2.0\resultui\dict\energy. Therefore, leaf Chl content is an important indicator of plant photosynthetic activity (Li et al., 2019). For example, in modern maize breeding, most grain yield increases have been primarily due to improved chloroplast structure and increased solar energy absorption by Chl (Li C. et al., 2015). Hence, Chl content is generally considered one of the major factors limiting photosynthetic efficiency (Hotta et al., 1997; Zhang et al., 2018). Solar energy capture by Chl has been known since as early as 1935 (Chlorophyll, 1935). However, gaps remain in the knowledge of Chl biosynthesis and the underlying genes in wheat. Hence, it is necessary to study the molecular basis of Chl biosynthesis in wheat to provide useful insight for improving photosynthetic efficiency. Further, Chl biogenesis is highly integrated with several critical metabolic networks, such as *de novo* fatty acid biosynthesis in the chloroplast (Wang and Benning, 2012), which not only influences plant cellular homeostasis but also impacts carbon fixation, assimilation, and distribution (Hölzl and Dörmann, 2019). Chl biosynthesis is also thought to take place upstream of a number of anabolic processes that regulate plant development, overall productivity, and adaptability to environmental change (Tripathy and Pattanayak, 2012).

**Figure 1:**
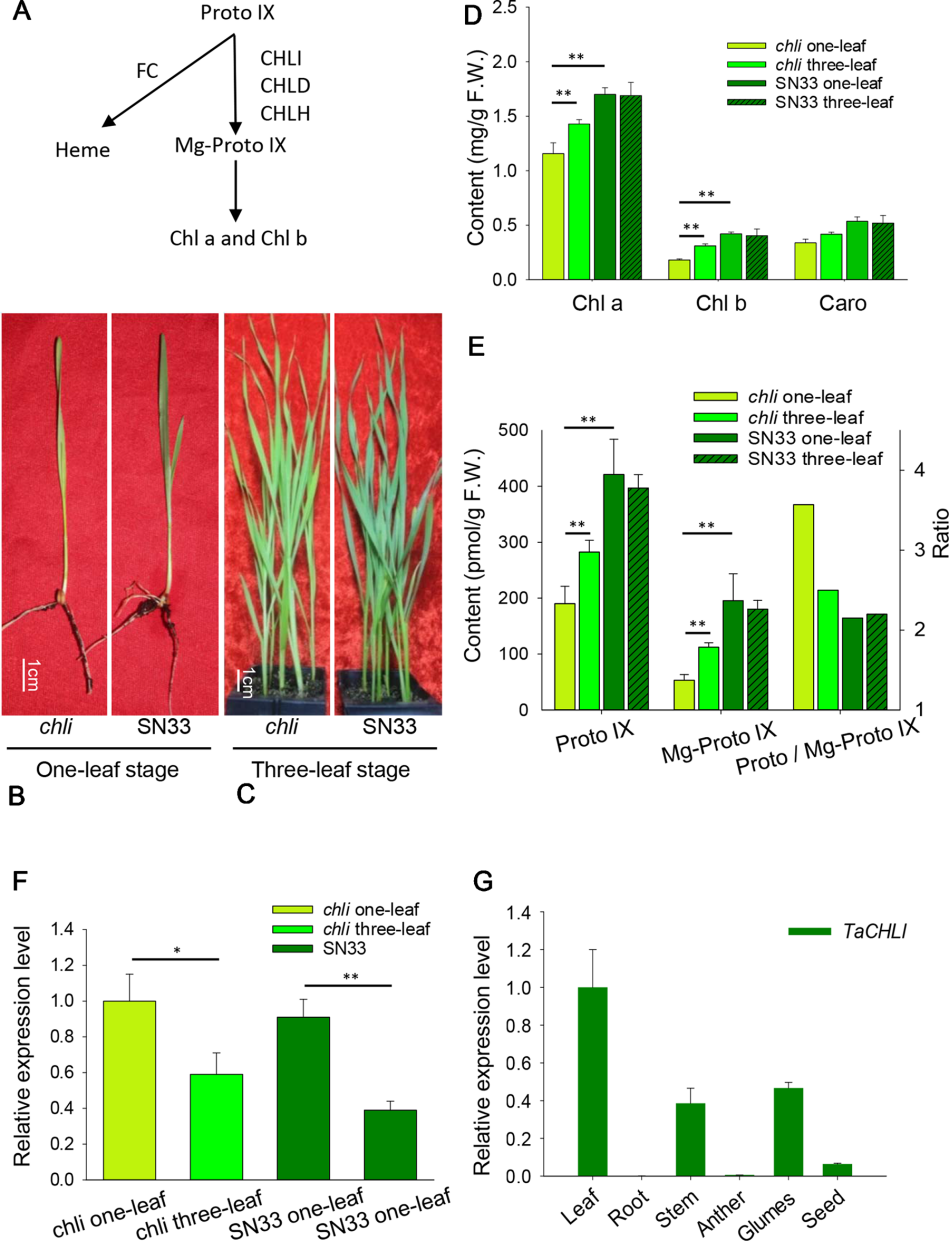
Characterization of *chli* and SN33. **(A)** Mg-Proto IX biosynthesis in chloroplasts. FC: protoporphyrin IX ferrochelatase. **(B)** Phenotype at the one-leaf stage. **(C)** Phenotype at the three-leaf stage. **(D)** Contents of Chl a, Chl b,and Caro at the one-leaf and three-leaf stages. **(E)** Content of Proto IX and Mg- Proto IX and the Proto IX to Mg-Proto IX ratio. **(F)** Relative expression of gene *TaCHLI* in *chli* and SN33. **(G)** Relative expression of gene *TaCHLI* in different tissues. Stem was from the green part; developing seed was from 20 days after pollination. Means and standard deviations were obtained from three independent replicates with three technical replicates. F.W.: fresh weight. ** indicates significant differences at p < 0.01. * indicates significant differences at p < 0.05.

Previous studies have found that Chl biosynthesis is a complex biological process involving at least 15 enzymatic reactions in Arabidopsis (*Arabidopsis thaliana*) (Tanaka and Tanaka, 2007). Specifically, a variety of enzymes and metabolites are involved in sequential reactions, which begin with glutamate formation and generate Chl a and Chl b as the end products (Beale, 2005; Tanaka and Tanaka, 2007). All genes involved in this process have been identified in Arabidopsis (Nasyrov et al., 1975; Tanaka and Tanaka, 2007). The insertion of metal ions (either Mg^2+^ or Fe^2+^) into Proto IX is not only the major branch point in the biosynthesis of Chl- and heme-derived pigments, but it is an important regulatory step in the Chl biosynthesis pathway (**Figure 1A**) (Tanaka and Tanaka, 2007). Insertion of Mg^2+^ into Proto IX is catalyzed by magnesium chelatase (Mg-chelatase) (Jensen et al., 1996a), which is an ATP-dependent reaction (Pardo et al., 1980; Lake et al., 2004). Mg-chelatase is a heterotrimeric enzyme complex that is composed of three subunits (i.e., I, D, and H) in photosynthetic bacteria (Bollivar et al., 1994) and plants (Nakayama et al., 1995). In particular, the CHLI subunit has been classified into the AAA^+^ (ATPases Associated with diverse cellular Activities) protein family according to its protein sequence and three-dimensional structure (Fodje et al., 2001; Lake et al., 2004). The CHLD subunit, which has an incomplete ATPase domain compared to CHLI, relies on the formation of an I-D complex through its interaction with CHLI to be stabilized (Hansson et al., 1999).

It is well known that the insertion of Mg^2+^ into protoporphyrin IX (Proto IX) is the most important step affecting Chl biosynthesis. Blocking this process may result in lowered Chl content and a green deficiency phenotype (Willows and Hansson, 2003). Chl deficient mutants have previously been reported in rice (Jung et al., 2003), cucumber (Gao et al., 2016), and maize (Sawers et al., 2006), all of which featured significantly decreased Chl content and pale-green leaves. The Mg-chelatase has been studied in these mutants. Particularly in rice, the mutant protein Oschli failed to interact with protein CHLD, which disrupted the biosynthesis of Mg-Proto IX. As a consequence, the Oschli mutant exhibited a yellow leaf phenotype with less Chl than wild-type Nipponbare (Zhang et al., 2015). Similarly, in cucumber C528, an amino acid substitution (G269R) occurred in subunit I protein CsChlI, which reduced Mg-chelatase enzymatic activity and resulted in a lower level of Chl and a golden-colored leaf phenotype (Gao et al., 2016). Similar mutants have also been described in barley (Jensen et al., 1996b). Presently, genes encoding subunits of Mg-chelatase have been characterized in a number of plants, but this complex remains to be further investigated in common wheat. The first observation of an albino wheat strain was made as early as 1929 (Smith and Harrington, 1929), but no genes related to leaf color have been identified to date. For example, wheat mutants ygld1, ygld2 (Li et al., 2013), and Ygm (Zhang et al., 2017) were only recently mapped to chromosomes. Not surprisingly therefore, Chl metabolism in common wheat is still unclear.

In this study, we explored the molecular mechanism underlying the wheat Chl-deficient mutant wheat *chli*, which had an amino acid mutation in the TaCHLI-7A protein (subunit I of the wheat Mg-chelatase) that resulted in decreased Chl content at the one-leaf stage and a pale-green leaf phenotype. Genetic analysis and yeast two-hybrid experiments were carried out to determine the differences between *chli* and wild-type Shaannong33 (SN33) in terms of Chl and lipid biosynthesis during wheat development. Previous studies have suggested that under saturating light conditions, photosynthetic rates in the modern wheat cultivar are associated with higher flag-leaf relative Chl content as compared with the landraces (Gaju et al., 2016). Moreover, Chl content can act as a proxy for leaf photosynthetic capacity (Inoue et al., 2016; Croft et al., 2017) and is intimately related to fatty acid biosynthesis, which is likely to influence wheat seedling development strongly. As a major regulatory point of Chl biogenesis, Mg-chelatase represents an important target for the improvement of photosynthetic efficiency (Zhang et al., 2018). Hence, our studies on *chli* open the door to a better understanding of Chl metabolism, photosynthetic regulation, and variety improvement in common wheat.

## Materials and Methods

### Plant Materials and Growth Conditions

The pale-green leaf mutant *chli* was selected from bread wheat cultivar Shannnong33 (SN33, dark green used as wild type) mutant library (Li M. et al., 2015) and self-crossed until F5. For genetic analysis, two F_2:3_ populations were produced from crossing *chli* × Zhongmai895 (ZM895, dark green) and ZM895 × *chli*, resulting in 190 and 211 individual lines. SN33, *chli*, and F2:3 populations were grown in plant incubator under a 16/8 h (day/night) cycle at a constant temperature of 22°C. Thirty seeds of each of the SN33, *chli*, and F_2:3_ lines were seeded in a 10 cm × 10 cm pot for phenotype observation. In the heading stage of SN33, flag leaves were cut and collected at 6:00, 7:00, 8:00, 9:00, 11:00, 13:00, 15:00, and 19:00 (temperature, degrees centigrade: 18, 20, 22, 24, 27, 24, 22, and 20, respectively; light and humidity was not controlled) from a greenhouse (a glass greenhouse with light from the sun to simulate field conditions) and kept in liquid nitrogen for further analysis.

### Content Measurement of Chl A, Chl B, Caro, Proto IX, and Mg-Proto IX

Chl pigments were measured at the one-leaf and three-leaf stage. Fully expanded leaf samples (0.2 g) were weighed into a 5-mL tube and ground into powder in liquid nitrogen, and 3 mL of 80% extraction buffer (water-acetone, 2:8, v/v) was then added. Each sample was soaked for 1 h in the dark. After extraction, the samples were centrifuged for 10 min at 12000 rpm. The supernatant was then filtered with 0.22-μm syringe filters (Organic-system, Sangon, China). We detected the absorbance value at wavelengths of 470, 645, and 663 nm using a spectrophotometer (SpectraMax M3; Molecular Devices, USA). The Chl and Caro contents were determined according to the equation of Lichtenthaler (Lichtenthaler, 1987). Agilent HPLC (High-Performance Liquid Chromatography) systems (Agilent 1260 Infinity II, USA) were used to determine the content of Proto IX and Mg-Proto IX (Mochizuki et al., 2008; Scharfenberg et al., 2015).

### Fatty Acid Measurement

The fatty acid was measured following Gas Chromatography (GC) measurement as described previously (Li et al., 2006; Yang et al., 2019). About 10 mg dry tissue powder was esterified in 2 mL methanol with 2.5% (v/v) H_2_SO_4_, and 50 μg triheptadecanoin was used as internal standard. The tube was then incubated at 80°C for 120 min. Thereafter, 1 mL of hexane and 2 mL 0.9% NaCl (w/v) were added to extract fatty acid methyl esters (FAMEs). FAMEs were quantified by GC as follows: 50°C for 1 min and ramped to 175°C at 35°C/min with a 1-min temperature hold, followed by a ramp to 230°C at 4°C/min, with a final 5-min temperature hold.

### DNA, RNA Extraction, and cDNA Synthesis

DNA of parent and individual lines was extracted by the CTAB method (Murray and Thompson, 1980), and total RNA was isolated by the TRIzol method (Couto et al., 2015). After extraction, the quality of the DNA and RNA was determined by gel electrophoresis using 1% agarose gel, and the concentration was measured with a spectrophotometer (SpectraMax M3; Molecular Devices, USA). gDNA-free RNA was reverse-transcribed to cDNA using the PrimeScript II 1st strand cDNA Synthesis Kit (Takara, Japan) according to the manufacturer’s protocol.

### Gene Mapping

The mutant gene was found to be located on chromosome 7A by using the wheat 660K SNP chip. To increase the accuracy and shorten the interval of the physical region, two DNA pools were made with the DNA from F_2_ (B23×ZM895), while individuals and phenotypic evaluation were from F_2:3_ lines. The DNA pool with recessive plants was composed of equal amounts of DNA from 40 homozygote pale-green plant samples. In contrast, the DNA pool with dominant plants was composed of equal amounts of DNA from 40 homozygote dark-green plant samples. The DNA of parental lines and two DNA pools were genotyped by using the 660K SNP wheat chip array. SNPs were processed with the Illumina Genome Studio Polyploid Clustering tool (v1.0) and mapped to the Chinese Spring wheat physical map (Appels et al., 2018). The distribution frequencies of polymorphisms (SNPs) on chromosomes were analyzed after SNP filtration using the following method: i. deleting the SNPs that were not detected in the Chinese Spring wheat physical map; ii. deleting the SNPs that were missing in one of two DNA pools; iii. deleting the SNPs that showed no polymorphism between parental DNA or the two DNA pools. The SNP distribution frequency ratio (polymorphism SNP numbers/total SNPs mapped to the wheat physical map) on the wheat chromosome physical map was calculated at 10 Mb physical intervals.

### Primer Design and Gene Cloning

In the high-frequency distribution region of polymorphism SNPs on a chromosome, candidate genes were predicted according to the open reading frame annotation from IWGSC RefSeq v1.0 (https://wheat-urgi.versailles.inra.fr/Seq-Repository/Annotations). Potential genes were selected according to the gene annotation and previous studies, such as genes related to chlorophyll synthesis, phytochrome et al. The full-length coding sequences of *TaCHLI-7A* and *Tachli-7A* were amplified using the primer pair CHLI-F/R (**Table 1**) from leaf cDNA of SN33 and *chli*, respectively. The PCR reactions were performed by using Pfu DNA polymerase (Sangon Biotech, China), and then PCR products were separated by 1% agarose gel electrophoresis. The DNA fragments were then cloned into a sequencing vector for sequencing after the DNA was recovered from the agarose gel. A Cleaved Amplified Polymorphic (CAP) primer pair, dCHLI-F/R, was designed according to the mutant site in Tachli-7A (dCHLI-F can only bind to the *TaCHLI-7A* DNA sequence specifically but not to *TaCHLI-7B* and *TaCHLI-7D*) (**Figure S1**). After PCR amplification using Pfu DNA polymerase (Sangon Biotech, China), the products were digested using restriction endonuclease Tth111I (NEB) and separated by 1% agarose gel electrophoresis. The PCR reaction was run as follows: 95°C for 90 s, followed by 32 cycles of 94°C for 30 s, 58-62°C (depending on primers) for 30 s, and 72°C for 3 min.

### Yeast Two-Hybrid Assay

Yeast two-hybrid analysis was performed using the GAL4 Two-Hybrid System according to the manufacturer’s instructions (Matchmaker; Clontech, USA). The full-length cDNA of *TaCHLI-7A* and *Tachli-7A* were cloned into the bait vector pGBKT7 and the prey vector pGADT7, respectively. Pairs of the plasmids BD and AD were co-transformed into yeast strain AH109 according to the manufacturer’s instructions. Transformants were first selected on plates containing a double-dropout SD medium (lacking Leu and Trp) and then tested on selective SD medium (lacking Leu, Trp, His, and Ade).

### Gene Transformation in *Arabidopsis* Mutant and Transgenic Plant Screening

In order to confirm whether *Tachli-7A* has normal function *in vivo*, the full-length coding sequences of *TaCHLI-7A* and *Tachli-7A* were cloned into pCambia1302 vector. The recombinant vectors pCambia1302-35s*::TaCHLI-7A* and pCambia 1302-35s*::Tachli-7A* were then transformed into the *Agrobacterium* strain GV3101. The GV3101-containing recombinant vector was then used to transform the *Arabidopsis* (Col-0) Chl-deficiency mutant (SALK_050029) homozygous lines through *Agrobacterium*-mediated transformation (Zhang X. et al., 2006). *Arabidopsis* Chl deficiency mutant SALK_050029 contains an insertion fragment at position 10203378 of chromosome 4 and shows pale-green leaves (https://abrc.osu.edu/stocks/number/SALK_050029). Transgenic lines were screened on 1/2 MS (Murashige and Skoog, 1962) plates supplemented with 25 mg L^-1^ hygromycin. In the presence of hygromycin screening medium, the positive transgenic plants exhibit roots and extend well on selective medium. By contrast, non-transgenic plants exhibit repressed root (Bent, 2006). Homozygous T_2_ plants of *TaCHLI-7A* were obtained, but transgenic plants, which contained wheat mutant gene *Tachli-7A*, did not recover the Chl in leaf, had weak growth (**Figure S10 A**), and could not survive in soil after two-week selection by hygromycin. Thus, no homozygous T_2_ plants of *Tachli-7A* were obtained. Multiple PCR was used to confirm the T_2_ transgenic plants. The PCR reaction contained 10 μM each of primers followed the manual of Pfu DNA polymerase (Sangon Biotech, China). The primer dCAP-F/R was used to confirm the transgenic *Arabidopsis*. Kan-F/R (**Table 1**), designed for aminoglycoside phosphotransferase (Kanamycin^+^ selection marker for bacteria, in plasmid but outside of the T-DNA region), was used to detect whether there was contamination of *Agrobacterium* that contained target plasmid. A plant tissue direct PCR Kit (OMEGA, USA) was used to confirm T_0_ *Tachli-7A* transgenic plants. The normal and mutant *Arabidopsis* used in the experiment were Col-0 type.

### Real-Time PCR Analysis

For Real-Time PCR analysis, primary cDNA was diluted 10 times, and 1 µl was used in a 20 µl Real-Time PCR reaction. PCR reaction and data collection were performed by using Applied Biosystems^®^ QuantStudio^®^ 3 (USA) with 2X SG Fast qPCR Master Mix (Low Rox) (Sangon Biotech, China). Relative gene expression levels were calculated by using the 2^-ΔΔCT^ method with three replicates (Miao et al., 2017). All data were normalized against the expression level of the wheat actin gene. All Real-Time PCR had three replicates with three technological replicates. Melting curves and the amplification efficiency of primers used for RT-qPCR (**Figures S2**–**S5**) were tested before the experiment. An 8-fold dilution of cDNA was made to measure Ct values and to generate standard curves. The slope, R^2^, and amplification efficiency were calculated by using SPSS (v17.0) (Zeng et al., 2017). For the *TaCHLI-7A* expression analysis of transgenic *Arabidopsis*, the primer pair actin-F/R from the ACTIN2 gene in *Arabidopsis* was used as the control gene (Lung et al., 2018).

### Statistical Analyses

The result for each sample is shown as mean ± standard deviation (SD) from three replicates. Two-tailed Student’s t-test was used to analyze the significance of differences between samples by using SPSS. P-value < 0.05 (*) and P-value < 0.01 (**) are regarded as significant.

## Results

### Phenotypic Characterization

Under plant incubator conditions, the wild type (SN33) showed dark green leaves (**Figures 1B, C**). In contrast, the mutant *chli* expressed pale-green leaves (**Figures 1B, C**). The F_2_ segregation of leaf color of crosses *chli* × Zhongmai895 and ZM895 × *chli* and also in the cross SN33 × *chli* is 3:1 (green: pale-green) (**Table 2**, **Figure S7** and **S8**). In order to explore the mechanism responsible for the pale-green leaves, pigment content was measured. Specifically, we compared SN33 and *chli* at the one-leaf stage and the three-leaf stage. Compared with SN33, the contents of Chl a, Chl b, and carotenoid (Caro) in *chli* were significantly lower, accumulating to 68.2%, 43.2%, and 62.5% of the levels observed in SN33 at the one-leaf stage, respectively (**Figure 1D**). At the three-leaf stage, the levels of Chl a, Chl b, and Caro in *chli* were 84.6%, 76.6%, and 80.4% of those in SN33, respectively. From the one-leaf stage to the three-leaf stage, increases in Proto IX and Mg-Proto IX were observed in both *chli* and SN33. In particular, a significant increase was observed in *chli*, with increases of 74.7% and 218.9% in Proto IX and Mg-Proto IX content, respectively. In contrast, Proto IX and Mg-Proto IX increased by 4.7% and -7.5% in SN33, respectively, from the one-leaf stage to the three-leaf stage. As shown in **Figure 3B**, Proto IX and Mg-Proto IX contents were consistently lower in *chli* than in SN33. At the one-leaf stage, Proto IX content in *chli* was only 32.8% of that in SN33, increasing to 60.0% at the three-leaf stage. For Mg-Proto IX, it was only 17.9% and 61.9%, respectively, at the one-leaf stage and the three-leaf stage. In SN33, the Proto IX to Mg-Proto ratio showed stable levels of 2.15 and 2.20 in the one-leaf and three-leaf stages. In contrast, the ratio was higher in *chli* in both stages but decreased from 3.75 to 2.50 from the one-leaf stage to the three-leaf stage (**Figure 1E**).

Meanwhile, we also explored the content of Proto IX and Mg-Proto IX and the Proto IX to Mg-Proto IX ratio in SN33 under simulated natural field conditions in a glass greenhouse during the flowering period. Interestingly, the content of Proto IX and Mg-Proto IX and the Proto IX to Mg-Proto ratio showed stable levels in response to changes in solar intensity, temperature, and humidity during the daytime. As shown in **Figure 6A**, under simulated field conditions, the contents of Proto IX and Mg-Proto IX between 6:00 to 19:00 were 1356 ± 102 pmol and 432 ± 29 pmol, respectively. The Proto IX to Mg-Proto IX ratio was 3.14 ± 0.08 under the same conditions. Furthermore, the contents of Proto IX and Mg-Proto IX and the Proto IX to Mg-Proto IX ratio were stable and climate-independent (**Figure 6A**). These values were, however, growth state-dependent. For example, under plant incubator conditions, the Proto IX to Mg-Proto IX ratio in SN33 was 2.16, but under simulated field conditions during daytime, the ratio was 3.14 on average.

### Altered Fatty Acid Biosynthesis in Leaves and Seeds

We next sought to explore the relationship between Chl deficiency and fatty acid content in leaves and seeds. The differences in total fatty acid content in leaves and seeds are shown in **Figure 8A**. At the one-leaf stage and three-leaf stage in both leaves and seeds, the total fatty acid content was significantly lower in *chli* than in SN33. Total fatty acid content in SN33 was 4.50 (% dry weight), 4.50, and 4.65 in seeds, leaves at the one-leaf stage, and leaves at the three-leaf stage, respectively. In *chli*, these values were 3.80, 3.71, and 3.61 (% dry weight). Remarkably, the fatty acid proportion was not significantly different between *chli* and SN33 (**Figures 8B–D**).

### Candidate Gene Isolation

**Figure 2:**
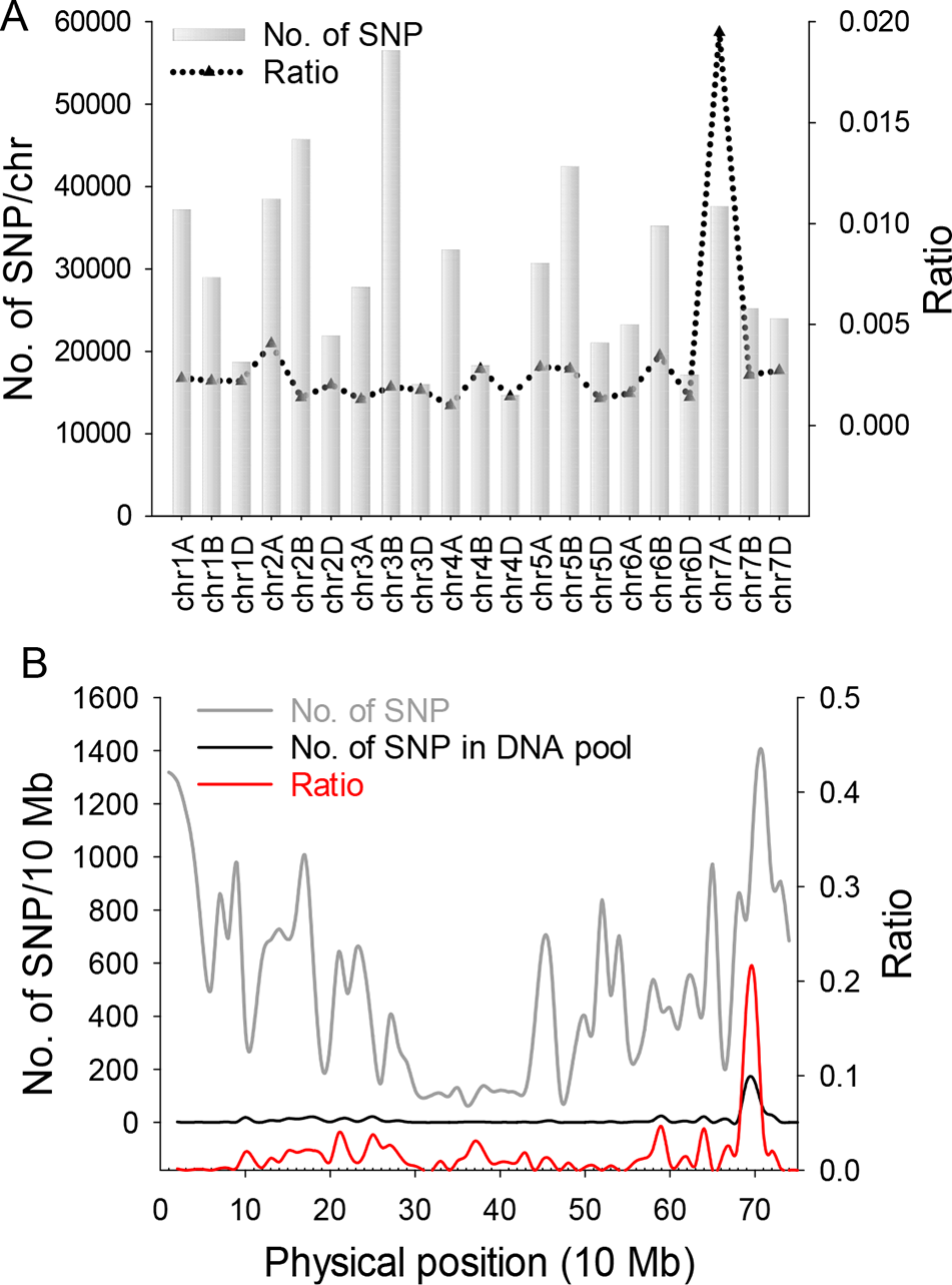
SNP distribution calculation and statistical analysis. **(A)** SNP distribution in 21 wheat chromosomes. Gray bars indicate the total number of SNPs on 21 wheat chromosomes from the SNP chip. Black triangles indicate the ratio value (polymorphism SNP numbers)/(total SNP numbers from chip). **(B)** Gray line represents the total number of SNPs from the wheat chip on chromosome 7A. Black line indicates the number of polymorphism SNPs on 7A from two DNA pools. Red line is the ratio value (polymorphism SNP numbers)/(total SNP numbers from chip) of 7A.

For genetic analysis, two F_2:3_ populations were generated by crossing *chli* × ZM895 and ZM895 × *chli*, which resulted in 190 and 211 individual lines (**Table 2**), respectively. Isolated DNA was hybridized on the wheat 660 K SNP chip array. After filtration, a total of 2010 polymorphic SNP probes were identified from two DNA pools and confirmed in the parental DNA. Those 2010 polymorphic SNP probes covered all 21 wheat chromosomes. Next, those 2010 polymorphic SNPs were mapped on the Chinese Spring wheat chromosomes based on IWGSC RefSeq v1.0 (**Figure 2A**). A total of 732 of the 2010 polymorphic SNPs were mapped on chromosome 7AL (**Figure 2B**). This distribution frequency of polymorphic SNPs indicated that the mutant gene was located on chromosome 7AL of Chinese Spring wheat. Meanwhile, the polymorphic SNP density distribution limited the mutant gene to a ~10 Mb region in the interval 670-680 Mb between SNP probes XA-111499939 and XA-109363735 (**Figure 2B**) (based on the Chinese Spring RefSeq v1.0 sequence) (**Figure 2B**). A total of 184 putative genes were predicted in this interval, from which the candidate gene TraesCS7A01G480700.1 (chr7A:672872418-672874726) was identified (**Table S1**). Potential candidate genes were selected on the basis of the gene annotation and previous studies, such as the genes that related to chlorophyll synthesis, phytochrome, et al. Interestingly, TraesCS7A01G480700.1-encoded protein CHLI in common wheat was found in this interval, which is a major subunit of Mg-chelatase, is involved in the biosynthesis of chlorophyll, and may correspond to the mutant gene of *chli*. Thereafter, TraesCS7A01G480700.1 was referred to as *TaCHLI-7A* and selected as a potential candidate gene for further analysis.

### Gene Sequencing and Marker Design

**Figure 3:**
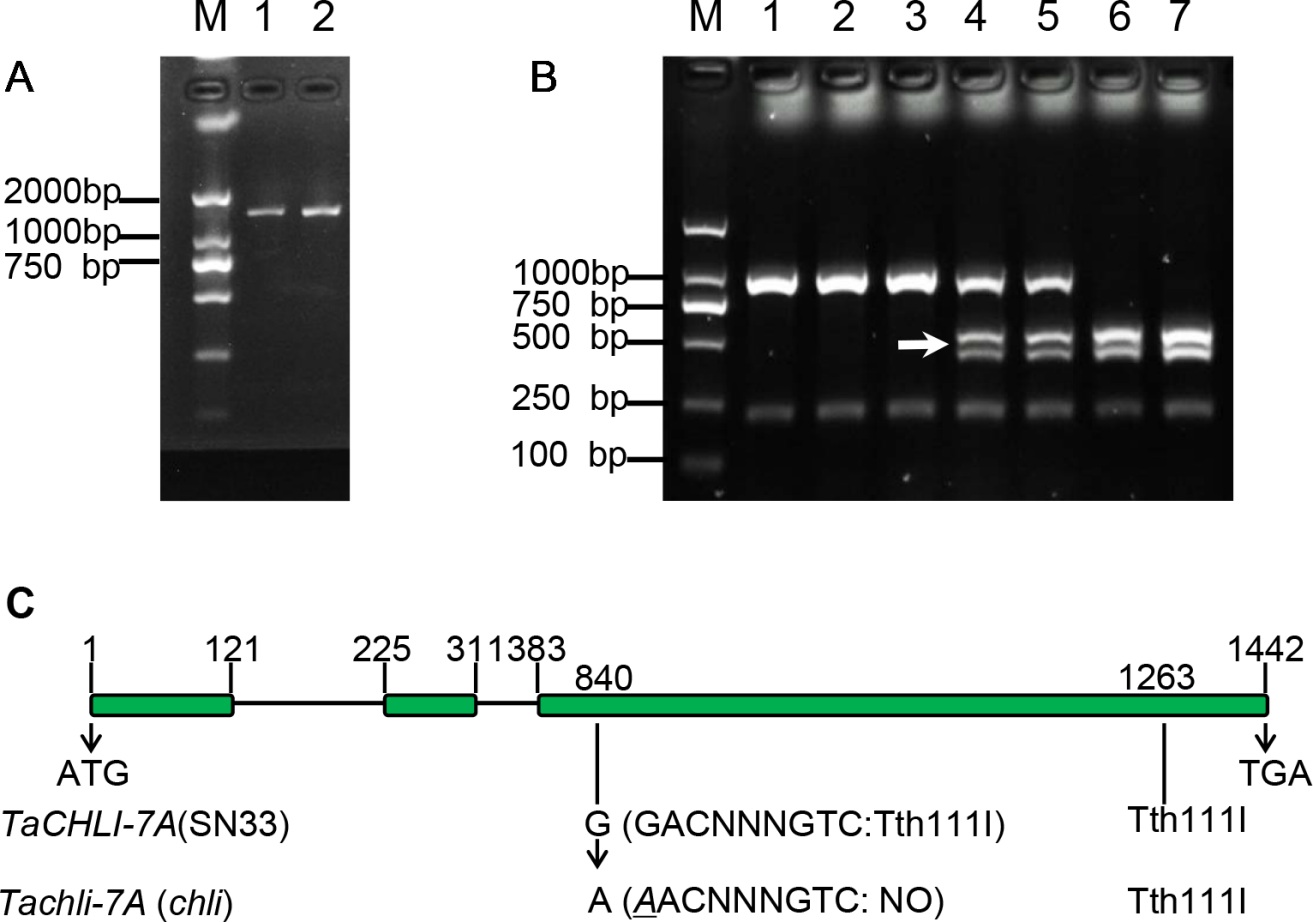
Characterization of gene *TaCHLI* of SN33 and *chli*. **(A)** Full-length cDNA production of *TaCHLI*. M: marker; 1: SN33 cDNA; 2: *chli* cDNA. **(B)** Discrimination by CAPS marker. M: DNA marker; 1: *chli*; 2 and 3: homozygotes (yellow-green leaf) from the F_2_ population; 4 and 5: heterozygotes (dark-green leaf) from the F_2_ population; 6: homozygotes (dark-green leaf) from the F_2_ population; 7: SN33. Arrow shows the restriction endonucleases cleavage site in *TaCHLI-7A*. **(C)** Structure and variations of *TaCHLI*. Electrophoresis was performed using 1% agarose gels.

**Figure 4:**
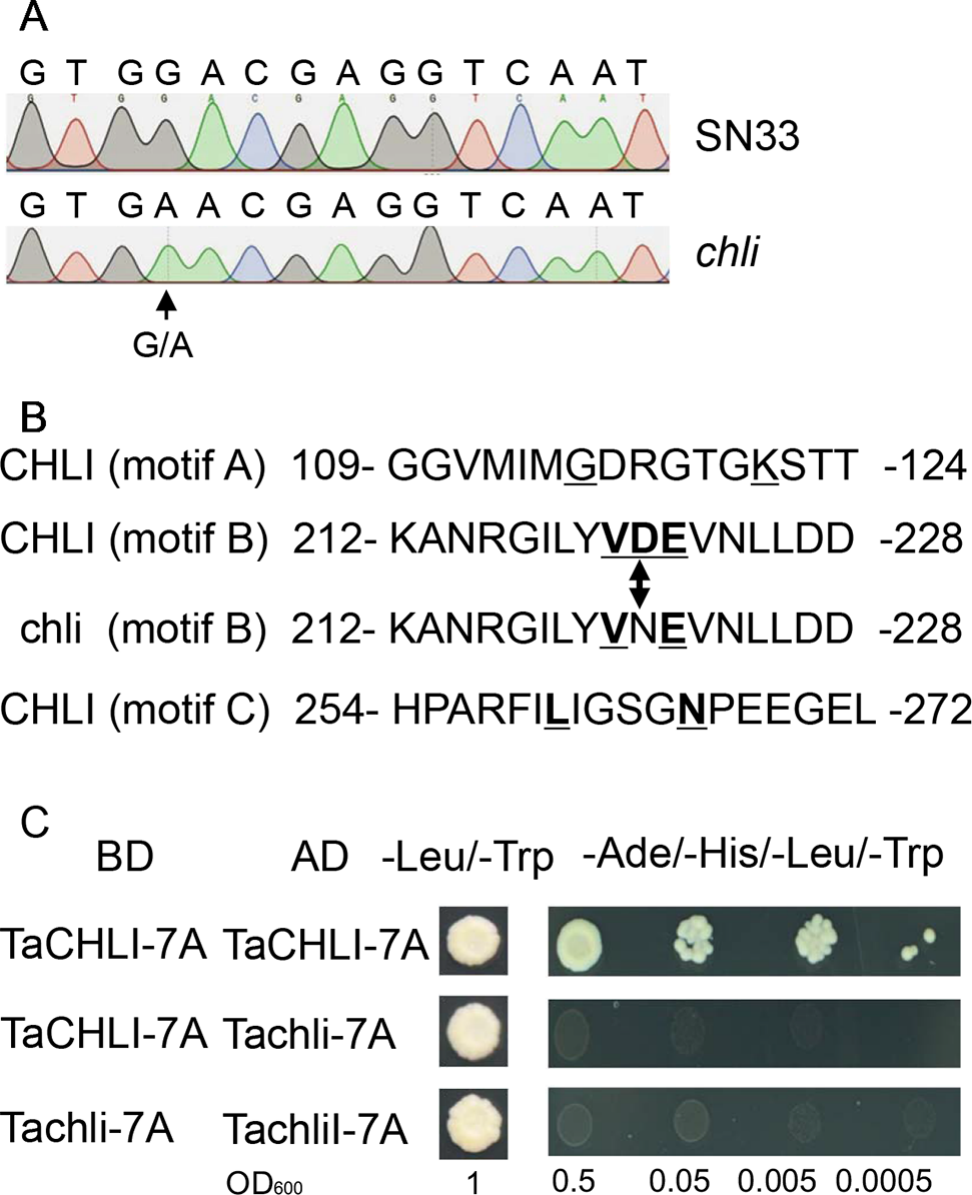
Sequencing results and amino acid residues. **(A)** Sequencing results of *TaCHLI-7A* and *Tachli-7A*. Black arrow indicates single-nucleotide variations. **(B)** Three conserved ATPase-binding motifs of TaCHLI. Bold and underlined letters indicate the conserved residues of CHLI in SN33. Double arrow indicates conserved substitutions from D (SN33) to N (*chli*). **(C)** Yeast two-hybrid assay between TaCHLI-7A and Tachli-7A. BD: pGBKT7; AD: pGADT7; -Leu/-Trp: SD medium lacking amino acids Leu and Trp. -Ade/-His/-Leu/-Trp: SD medium lacking amino acids Ade, His, Leu, and Trp.

Based on the sequence of gene TraesCS7A02G480700.1 (**Figure S6**), the primer pair CHLI-seqF/R (**Table 1**) was designed to amplify the full-length cDNA of *TaCHLI* from SN33 and *chli*. As shown in **Figure 3A**, a 1266 bp-length cDNA sequence was obtained. Simultaneously, the sequencing results also revealed one single nucleotide substitution in *TaCHLI-7A* between SN33 and *chli* (**Figure 4A**). The nucleotide G in SN33 at position 664 was replaced by A in *chli*, which resulted in a single amino acid substitution (D221N) (**Figure 4B**). In parallel, it was found that this mutation did not occur in other normal wheat cultivars. All of the tested homozygote pale-green plants of F_2_ contained an A at this nucleotide site. In contrast, the tested homozygote green plants of F_2_ had a G while heterozygote green plants of F_2_ had an A:G genotype. The primer pair dCHLI-F/R (**Table 1**) was then used to amplify part of the gene sequence of *TaCHLI-7A* and *Tachli-7A* specifically. Meanwhile, *TaCHLI-7B* on chromosome 7B and *TaCHLI-7D* on chromosome 7D were not amplified by dCHLI-F/R. Sequencing analysis of *TaCHLI-7A* revealed a Tth111I recognition site (GACNNNGTC) in the PCR products of SN33 (5′-GACNNNGTC-3′). However, the Tth111I recognition site in the PCR products of mutant *chli* (5′-AACNNNGTC-3′) was impaired by the nucleotide replacement from G (SN33) to A (*chli*) at position 664. Following the PCR amplification, the product was digested using the restriction endonucleases Tth111I (NEB). The PCR product from SN33 (GACNNNGTC) could be digested, while the product from *chli* (AACNNNGTC) could not (**Figure 3B**). Therefore, the primer pair dCHLI-F/R can be used to easily distinguish the genotype of *chli* from that of the wild type.

**Table 1:** Primers used for PCR and qPCR.

Name	Sequence (5’-3’)
CHLI-F	GTGTCTCCCAATCCCTCTC
CHLI-R	ACCTCGAGAGTAATCTAGCCA
dCHLI-F	TGTCCTGCCTACAATGCGGTA
dCHLI-R	ACCTCGAGAGTAATCTAGCCA
qCHLI-F	TCACCACCACCAAGATCACCA
qCHLI-R	CTCGAACGCCTTGACACCTTC
TaActin-F	TCAGCCATACTGTGCCAATC
TaActin-R	CTTCATGCTGCTTGGTGC
Kan-F	ACGGAAGGAATGTCTCCTGCTA
Kan-R	TCCTTCCAGCCATAGCATCATGT

### Yeast Two-Hybrid Assay and Transformation of the *Arabidopsis CHLI* Mutant

**Figure 5:**
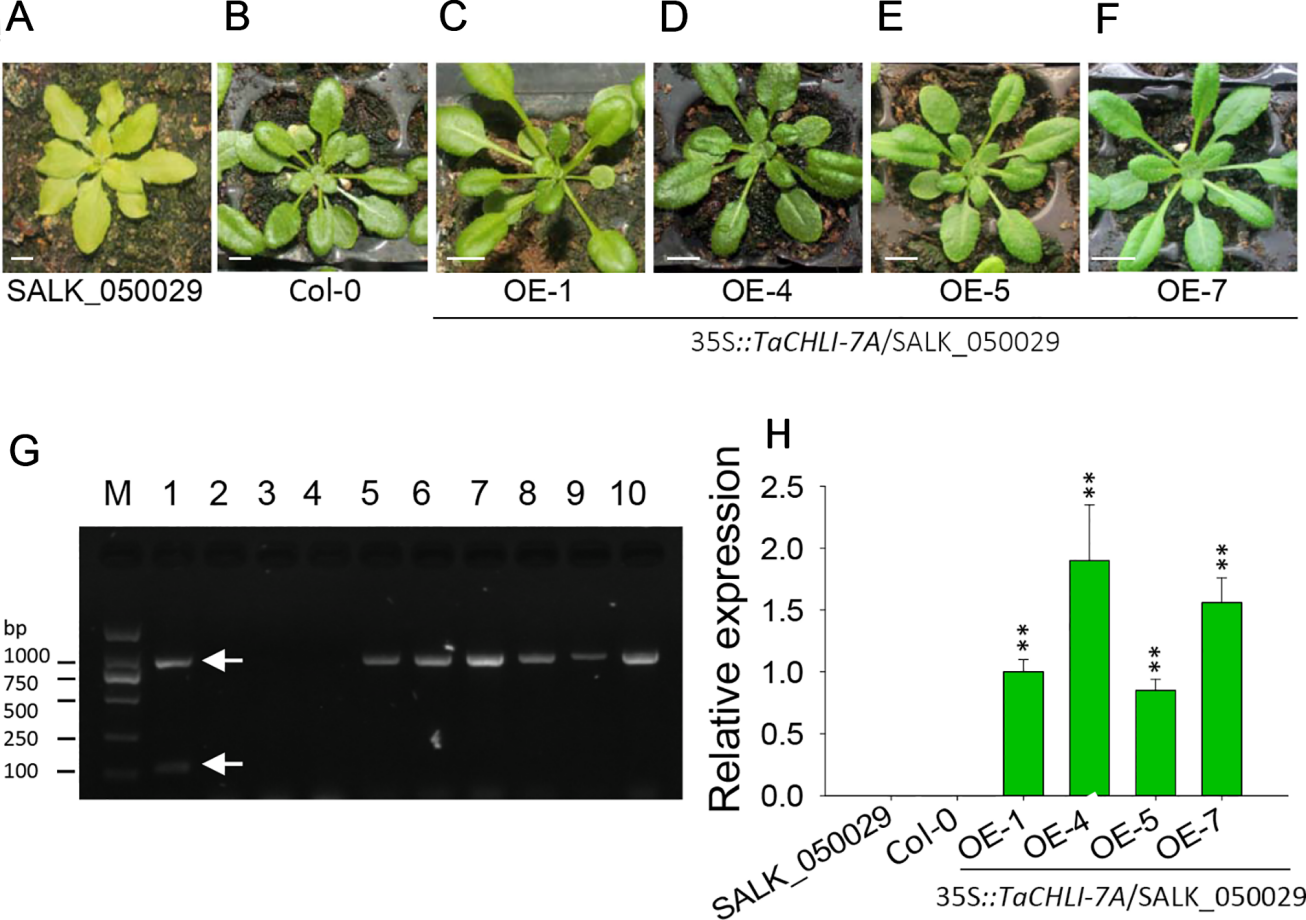
Phenotype of homozygous T_2_ transgenic plants of *Arabidopsis* SALK_050029 mutant. **(A)** 3-week-old mutant SALK_050029. **(B)** 3-week-old *Arabidopsis* (Col-0). **(C–F)** 3-week-old homozygous T_2_ of 35S*::TaCHLI-7A* transgenic SALK_050029 mutant plant. **(G)** PCR results of transgenic SALK_050029 of 35S*::TaCHLI-7A* gene. M: DNA Marker; PCR templates of Lane 1-4: plasmid of 35S*::TaCHLI-7A*, water, DNA of SALK_050029, DNA of Col-0; 5 and 6: tissue of T_0_ of 35S*::TaCHLI-7A* transgenic SALK_050029; 7-10: DNA of OE-1, OE-4, OE-5, OE-7 respectively. **(H)** Relative expression level of gene *TaCHLI-7A* in SALK_050029, Col-0 and transgenic *TaCHLI-7A* SALK_050029. Significant differences of the expression level were calculated by comparing WT, OE-1, OE-4, OE-5, and OE-7 with the expression level value of SALK_050029 (the value was marked as 0), calculating the expression level of OE-1 as 1. OE is overexpression. Means and standard deviations were obtained from three independent replicates with three technical replicates. ** indicates significant differences at p< 0.01. Bars: 1 cm. Higher white arrow in G indicates the PCR product of dCAP-F/R; lower white arrow in E indicates the PCR product of Kan-F/R.

We cloned *TaCHLI-7A* and *Tachli-7A* into bait vector pGBKT7 and prey vector pGADT7, respectively, and the yeast two-hybrid system was used to test the interaction between them. The results showed that yeast cells that contained vector pGBKT7-*TaCHLI-7A* and pGADT7-*TaCHLI-7A* could not only grow on medium SD (-Leu/-Trp) but also on medium SD (-Ade/-His/-Leu/-Trp), which suggested that protein TaCHLI-7A could interact with itself (**Figure 4C**). However, yeast cells that contained vector pGBKT7-*TaCHLI-7A* and pGADT7-*Tachli-7A* could not grow normally on medium SD (-Ade/-His/-Leu/-Trp) (**Figure 4C**). Furthermore, yeast cells with vector pGBKT7-*Tachli-7A* and pGADT7-*Tachli-7A* did not grow normally on medium SD (-Ade/-His/-Leu/-Trp) (**Figure 4C**). In general, normal TaCHLI-7A protein could interact with itself, but it could not interact with mutant protein Tachli-7A. In addition, mutant protein Tachli-7A could not interact with itself. As shown in **Figure S10A**, the *Arabidopsis* mutant SALK_050029, which contains a mutation in the *CHLI* gene, exhibited pale-green leaves. We next transformed *35s::TaCHLI-7A* and *35s::Tachli-7A* constructs in this *Arabidopsis* mutant to confirm the function of the wheat genes. At the T_2_ generation, the SALK_050029 mutant carrying *35s::TaCHLI-7A* showed a green leaf phenotype (**Figures 5C–F**) to varying degrees. In contrast, SALK_050029 carrying *35s::Tachli-7A* showed a pale-green phenotype (**Figure S10A**) and could not survive in soil after selection by hygromycin. In the T_2_ generation, the content of Chl in homozygous transgenic plants was significantly higher than that in *Arabidopsis* SALK_050029 mutant (**Figure S9**), which suggested that the specific mutation in Tachli-7A impaired Mg-chelatase activity.

### Expression Pattern of *TaCHLI*

**Figure 6:**
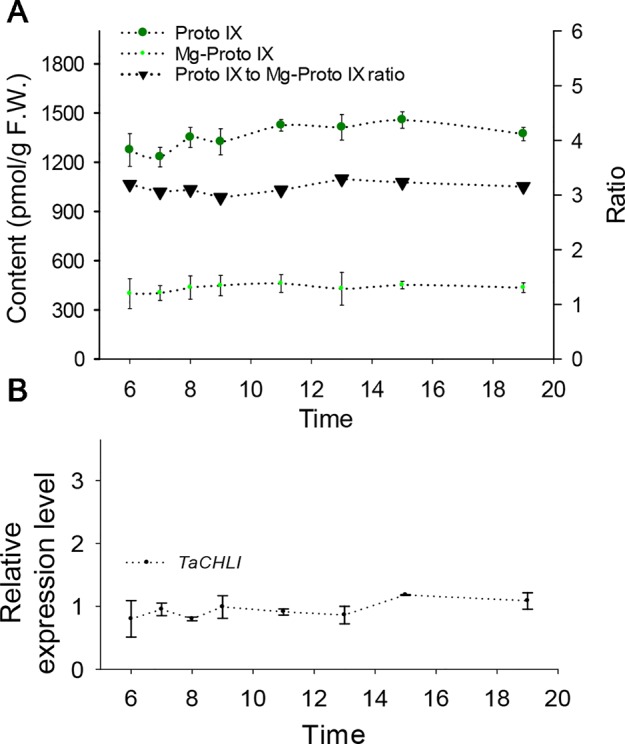
Contents of Proto IX and Mg-Proto IX, Proto IX to Mg-Proto IX ratio, and expression level of *TaCHLI* in SN33 under simulated field conditions. **(A)** Contents of Proto IX and Mg-Proto IX and Proto IX to Mg-Proto IX ratio. **(B)** *TaCHLI* expression levels under simulated field conditions from 6:00 to 19:00. Means and standard deviations were obtained from three independent replicates with three technical replicates. F.W.: fresh weight.

We next explored the expression pattern of *TaCHLI* by using RT-qPCR. Melting curve analysis of the real-time PCR products revealed a single peak (**Figures S2** and **S4**), which confirmed the specificity of the primers. In addition, the efficiency of the primer qCHLI-F/R was 96.8%, with an R^2^ is 0.99 (**Figure S3**). For *TaActin*, the primer efficiency was 99.7%, with an R^2^ is 0.99 (**Figure S5**). High specificity and amplification efficiency indicated that reliable results could be obtained from this experiment. As shown in **Figure 1G**, mRNAs were most abundant in green tissues (e.g., leaves, glumes, and stems). As expected, few mRNAs could be detected in anthers and roots, which lack chloroplasts. In developing seeds, the expression level was higher than that in anthers and roots but lower than that in green tissues. These results suggested that *TaCHLI* was mainly expressed in green tissues. Therefore, we chose to only examine the expression pattern of *TaCHLI* in leaves of SN33 and *chli*. As shown in **Figure 1F**, the expression level of *TaCHLI* in *chli* was higher at the one-leaf stage than that at the three-leaf stage. A similar expression pattern was observed in SN33. Flag leaves of SN33 were collected during the flowering period in order to assess the expression pattern under field conditions. Meanwhile, we found that the expression of *CHLI* in SN33 was quite stable during the daytime (**Figure 6B**).

## Discussion

**Figure 7:**
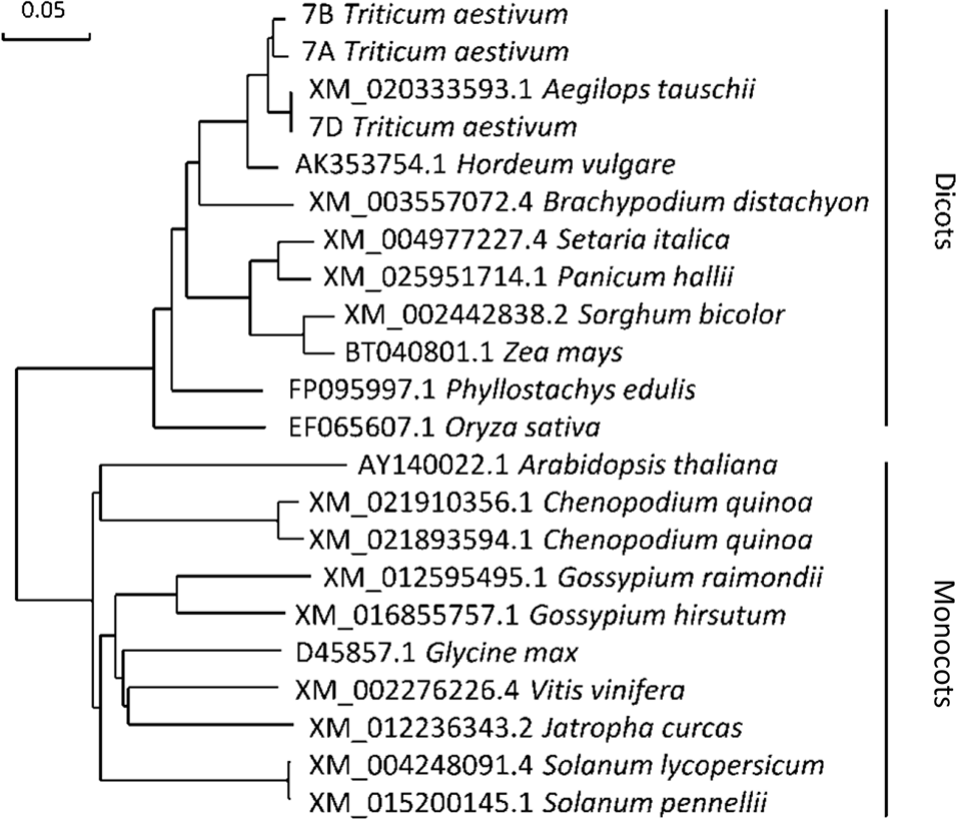
Molecular phylogenetic analysis of gene *CHLI*. The number before the Latin name represents the accession number from the National Center for Biotechnology Information (NCBI). 7A, 7B, and 7D represent the cDNA sequences from Chinese spring wheat. Phylogenetic analysis of gene *CHLI* conducted by using the maximum likelihood method and bootstrap confidence values exceeding 80% from 1000 replicates.

Previous studies have identified various leaf color mutants in common wheat (Williams et al., 1985; Zhang et al., 2017). However, only a few genes have been characterized, especially in common wheat, to date (Zhang et al., 2017). In this study, we identified a pale-green leaf mutant with Chl deficiency in the seedling stage. Genetic analysis indicated that the pale-green leaf phenotype was linked to a pair of recessive nuclear genes (**Table 2**), which is consistent with the previous reports that most of the Chl deficiency genes are recessive (Willows and Hansson, 2003). The 660K wheat SNP chip, which is generally accepted as an efficient tool for gene mapping (Wu et al., 2018), was used to localize the mutant gene to a 670-680 Mb region of wheat chromosome 7AL. In this region (**Figure 2B**), gene TraesCS7A02G480700.1 was predicted as the candidate gene and was named *TaCHLI-7A*. Interestingly, this gene shared high homology with the *Arabidopsis* gene *CHLI* 1 (Apchelimov et al., 2007; Kim et al., 2009) (**Figure 7**). Sequence analysis further revealed a single-nucleotide mutation in *TaCHLI-7A* at position 664, which changed G (in SN33) to A (in *chli*) (**Figure 4A**).

**Table 2:** Genetic analysis of leaf color in progenies derived from the crossings *chli*×ZM895 and ZM895×*chli* at the seedling stage in greenhouse.

Crossing	Parents and generations	No. of plants			Expected ratio	χ^2^	P
		YG.	Seg.	DG.			
*chli*×ZM895	*chli*	5		0			
	ZM895	0		5			
	F_1_	0		20			
	F_2_	43		168	1:3	0.051	0.82
	F_2:3_	40	111	54	1:2:1	0.075	0.96
ZM895×*chli*	*chli*	5		0			
	ZM895	0		5			
	F_1_	0		20			
	F_2_	53		180	1:3	0.011	0.92
	F_2:3_	47	96	53	1:2:1	0.009	0.99
SN33×*chli*	*chli*	5		0			
	SN33	0		5			
	F_1_	0		30			
	F_2_	35		86	1:3	0.029	0.86

YG, yellow-green; DG, dark-green; Seg, segregation; F_2:3_: from individual of F_2_; χ_20.01_(1)=6.63; χ_20.01_(2)=9.21.

CHLI is a highly conserved protein in plants (Zhang et al., 2018). As shown in the phylogenetic tree (**Figure 7**), CHLI from dicots tended to cluster together, which was also observed for monocots. Being highly conserved in protein implies that protein CHLI plays a crucial role in the determining of the Mg-chelatase activity. Previous studies indicated that CHLI belongs to a superfamily of putative DNA-dependent ATPases and contains three highly conserved motifs (i.e., motifs A, B, and D) (Walker and Willows, 1997). In mutant *chli*, the aspartic acid (D) in SN33 at position 221 was replaced by asparagine (N), which occurred on motif B of the CHLI (**Figure 4B**). Gao et al. found that the mutation in amino acid of CHLI in Cucumber resulted in Chl deficiency in plant leaves (Gao et al., 2016). This was confirmed in rice mutant *chlorina-9* (Zhang H. et al., 2006), maize mutant *Oy1* (Sawers et al., 2006), soybean mutant *yll* (Campbell et al., 2015). In short, amino acid substitution in the conserved motif may impair the enzyme activity of Mg-chelatase and then block the biosynthesis of Chl a and Chl b. This result was confirmed by an *Arabidopsis* leaf Chl-deficiency recovery experiment that showed that TaCHLI-7A could recover leaf Chl deficiency to a normal green leaf phenotype but that Tachli-7A did not function in leaf Chl-deficiency mutant SALK_050029. Meanwhile, *chli* is the first mutant in which the mutation occurred on the conserved amino acid residue in motif B.

**Figure 8:**
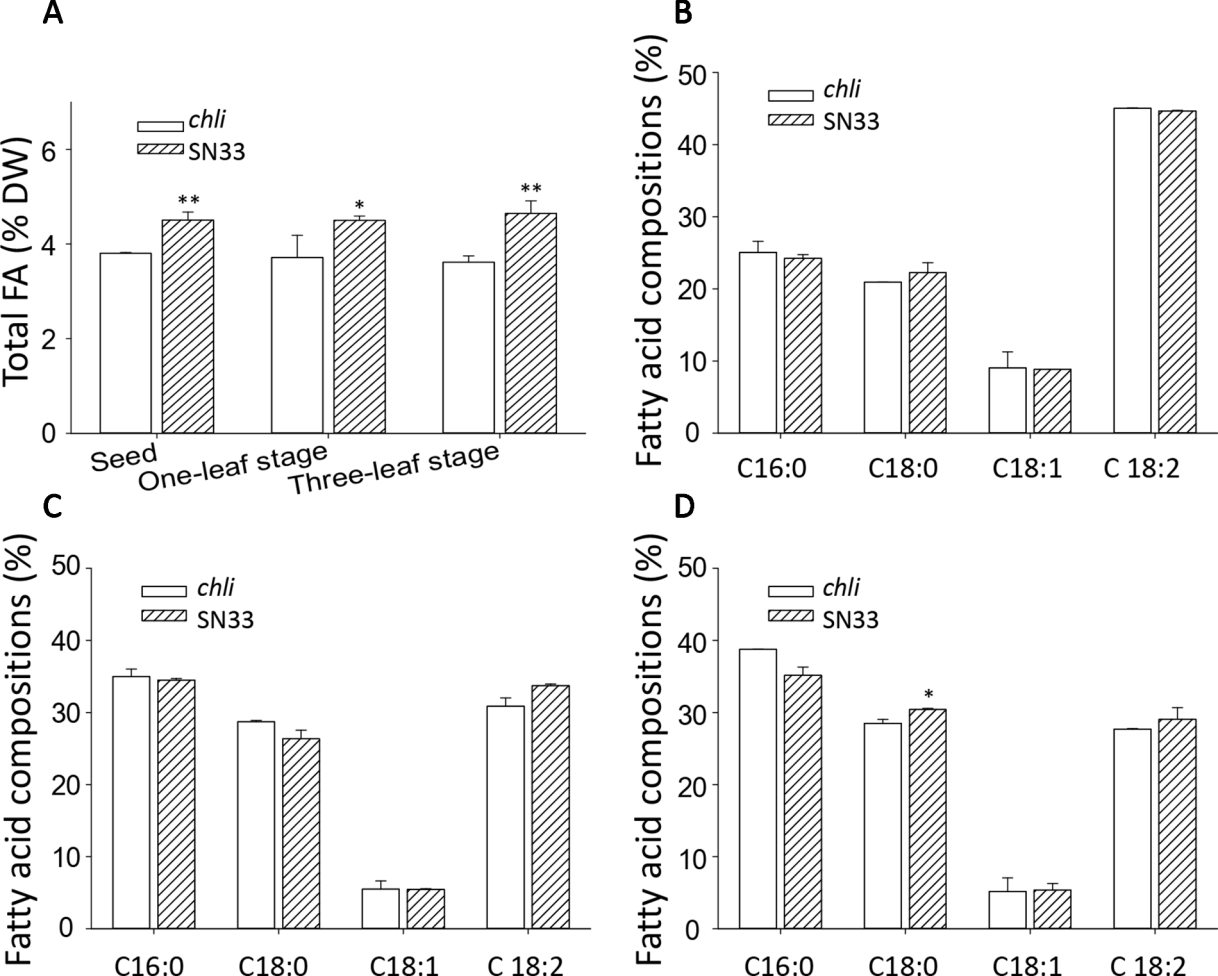
Fatty acid content of wheat. **(A)** Total fatty acid content of seeds and leaves. **(B)** Fatty acid compositions of leaves at the one-leaf stage. **(C)** Fatty acid compositions of leaves at the three-leaf stage. **(D)** Fatty acid compositions of seeds. FA: fatty acid. SN33/*chli*: ratio of average content of SN33 to *chli*. Means and standard deviations were obtained from three independent replicates with three technical replicates. ** indicates significant differences at p < 0.01. * indicates significant differences at p < 0.05.

Fatty acids play an important role in plant development, not only as one of the major constituents of cellular membranes but also as signal transductors in plant responses to various abiotic/biotic stresses (Zhang et al., 2005; Upchurch, 2008). Despite the fact that many studies have been carried out to identify factors that affect fatty acid biosynthesis in plants (Williams et al., 1988; Baud and Lepiniec, 2009; Luisa Hernandez et al., 2011), little is known about the relationship between Chl synthesis and fatty acid biosynthesis. Our study demonstrated that the total lipid content of both leaves and seeds (**Figure 8A**) was significantly lower in mutant *chli* than that in SN33. As plastids are the dominant site for the anabolic production of nascent lipids (Hölzl and Dörmann, 2019), large amounts of acetyl-CoA are needed (Lichtenthaler, 1999). Meanwhile, phytol, part of the Chl structure, shares a common biosynthetic precursor substrate with lipids in biosynthesis (Lichtenthaler, 1999). Additionally, photosynthesis in chloroplasts generates the ATP for fatty acid biosynthesis (Slabas and Fawcett, 1992). Hence, Chl deficiency in mutant *chli* may restrict the efficient production of ATP, thereby altering fatty acid production. Further exploration is necessary to better understand the relationship between Chl and fatty acid biogenesis, which may ultimately provide insight for improving wheat breeding and enhancing wheat grain quality (Wang et al., 2011).

Chl biosynthesis is a complex process that requires a series of enzymes. Mg-chelatase-catalyzed insertion of Mg^2+^ into Chl biosynthesis is the branching point in the biosynthesis of Chl and heme as well as being a putative critical step in Chl biosynthesis (**Figure 1A**) (Moulin and Smith, 2005). For example, rice Chl-deficiency mutant *ell* (CHLI mutant in rice) exhibited a 4.7 times higher Proto IX to Mg-Proto IX ratio than wild-type plants (Zhang et al., 2015). In our study, this ratio in *chli* (3.37 and 2.50 at the one-leaf and three-leaf stages, respectively) was higher than that in SN33 (2.15 and 2.20 at the one-leaf and three-leaf stages, respectively) (**Figure 1E**). The higher ratio of Proto IX to Mg-Proto in *chli* demonstrated that metabolic substrate conversion efficiency was blocked in *chli*. These results further confirmed that the function of Tachli-7A was impaired in *chli*. It should be noted that the content of Proto IX was lower than that in SN33. This result is different from *ell*, which showed higher levels of Proto IX content than wild type (Zhang H. et al., 2006). Interestingly, some reports have indicated that mutants blocked at CHLI do not show detectable increases in Chl intermediates (Papenbrock et al., 2000). Meanwhile, we also found that the ratio of Proto IX to Mg-Proto in SN33 was leaf development-independent (2.15 and 2.20 at the one-leaf and three-leaf stages, respectively) (**Figure 1E**), as well as being steady at around 3.14 under simulated field conditions during the daytime (**Figure 6A**). Taken together, these results demonstrated that mutation of CHLI blocked the conversion of Proto IX to Mg-Proto IX in plants. Meanwhile, different molecular mechanisms may operate in different plants to regulate Mg-chelatase activity and balance the Proto IX to Mg-proto IX ratio. More attention should be paid to this subject.

Previous studies on the self-assembly of CHLI demonstrated that the aggregated CHLI protein complex contains 6-8 CHLI subunits (Jensen et al., 1998). In the initial step of Mg-chelatase activation, the CHLI protein complex interacts with one D subunit initially (Walker and Willows, 1997), which suggests that CHLI self-assembly is one necessary step for the Mg-chelatase. Therefore, analyzing the ability to form the CHLI protein complex provides a potential way to confirm the function of CHLI. Yeast two-hybrid assay demonstrated that the mutant protein Tachli-7A may not be able to form the protein complex, at least in the yeast two-hybrid system. Furthermore, Tachli-7A may lose the ability to interact with the D subunit. A similar result was found in the etiolated leaf and lethal mutant *ell* of rice, where the malfunctioned protein OSchli lost the ability to interact with OsCHLD, further leading to reduced contents of Mg-Proto IX and Chl (Zhang et al., 2015). It is consistent with our results that normal TaCHLI-7A protein can restore the Chl deficiency in mutant SALK_050029 (**Figure 5A**) but Tachli-7A cannot (**Figure S10A**). Hence, we infer that mutation on Tachli-7A is an important potential contributor to pale leaf formation in *chli*.

A clear expression pattern of *TaCHLI* contributes to an improved understanding of the characteristics of Chl biosynthesis. Our results indicated that the expression of *TaCHLI* showed the highest levels in leaves, was lower in developing seeds and stems, and was negligible in roots and anthers of SN33 (**Figure 1G**). This is consistent with previous studies in cucumber (Gao et al., 2016) and soybean (Zhang et al., 2018). As a major staple food (Paux et al., 2008), the growth of wheat under field conditions is influenced by many factors, such as light and temperature. Hence, it is necessary to determine how climate factors influence TaCHLI expression, as well as the content of Proto IX and Mg-Proto IX. The results (**Figure 6B**) suggested that the expression level of *TaCHLI* and the Proto IX to Mg-Proto ratio in SN33 was steady in the daytime, but interestingly, the Proto IX to Mg-Proto ratio was higher under simulated field conditions (3.14) than in a plant incubator (2.16). These results indicated that the conversion efficiency from Proto IX to Mg-Proto is also growth-state dependent and was not affected by climate factors to a certain extent. More studies should be done to clarify the unknown mechanism behind it.

## Data Availability Statement

The raw data supporting the conclusions of this article will be made available by the authors, without undue reservation, to any qualified researcher.

## Author Contributions

CSW conceived the original research. CJW performed most of the experiments and wrote the article with LZ. CJW and LZ contributed equally. YL, YX, and NW prepared the plant materials and measured the phenotype. ZB modified the manuscript.

## Funding

This project was partially supported by the National Key Research and Development Program of China (Project: 2016YFD0102101) and by the Construction Project of Nanyang Wheat Experimental Demonstration Station of Northwest A&F University (Project: A289021417).

## Conflict of Interest

The authors declare that the research was conducted in the absence of any commercial or financial relationships that could be construed as a potential conflict of interest.
