# Supplementary material for: Single Nucleotide Mutagenesis of the TaCHLI Gene Suppressed Chlorophyll and Fatty Acid Biosynthesis in Common Wheat Seedlings
Source: Front Plant Sci. 2020 Feb 20;11:97. doi: 10.3389/fpls.2020.00097 (PMC7046076; doi:10.3389/fpls.2020.00097)
Supplement: Supplementary file 1 [file DataSheet_1.pdf]

Genetic and physiological analysis of a wheat (*Triticum aestivum* L.) pale-green leaf mutant *chli*

dCHLI-F: TGTCCTGCCTACAATGCGGTA
TaCHLI-7A: TGTCCTGCCTACAATGCGGTACTGTTTACT.CTCAT
TaCHLI-7B: TGTCC**AA**CC**C**ACTATG**TTC**T**G**CAGGCTCTGACGTAT
TaCHLI-7D: TGTCC**AA**CC**C**ACAATG**TTC**T**G**CTGGCTCTGACGTAT

Figure S1. Primer design of dCHLI-F. Due to the difference of DNA sequence, primer dCHLI-F: TGTCCTGCCTACAATGCGGTA could combine with the DNA sequence of TaCHLI-7A: TGTCCTGCCTACAATGCGGTACTGTTTACT.CTCAT.

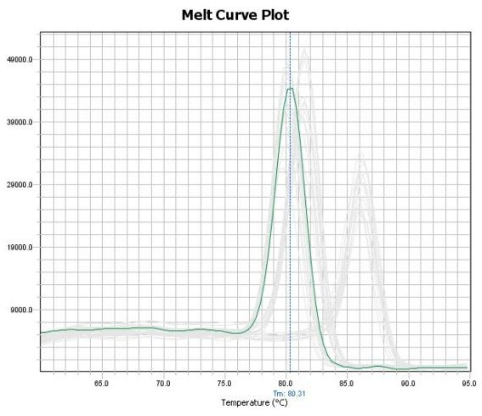

Figure S2.The melt curve plot of TaActin-F/R

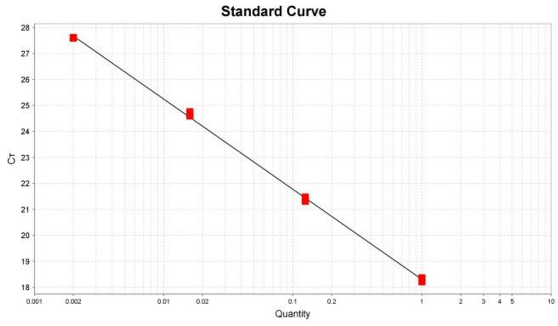

Figure S3.The standard curve of TaActin-F/R. slope=-3.473, R^2=0.999, efficiency=99.7%.

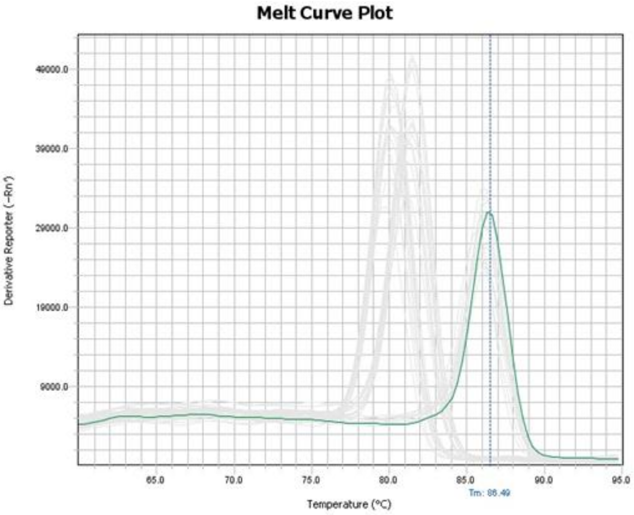

Figure S4.The melt curve plot of qCHLI-F/R.

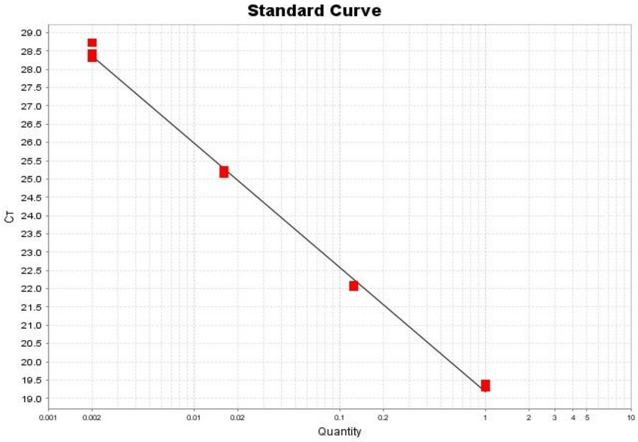

Figure S5.The standard curve of qCHLI-F/R. slope=-3.401, R2=0.997, efficiency=96.8%.

>TraesCS7A02G480700.1(*TaCHLI-7A*))
ATGGCCATGGCCTCCCCGTTCTCCCAGGCGTCGGCCGCCGCCGCCTCGCCGGCCCTCCCCTTCTC
CGTCTCCACCTCCCGCCCTCTCTCCCTCACCACCGCCGCAACCGCCGCCGTCTCAGCCCGGGCTC
CGTGCAGGGGCAGCAGAGGATTCCGCCGCGGCCGCTTCGCCGTCTGCAATGTCGCTGCCCCCTC
CGCCGCCGAGCTGGAGACCAAGCCGGCGGCGGCCGCGAAGGAGAGCCAGCGGCCGGTGTACCC
GTTCCCGGCGATCGTGGGGCAGGACGAGATGAAGCTCTGCCTGCTGCTCAACGTCATCGACCCC
AAGATCGGCGGCGTCATGATAATGGGCGACCGGGGCACCGGCAAGTCCACCACCGTCCGCTCCC
TCGTCGACCTGCTCCCGGACATCAGCGTCGTGGTCGGCGACCCGTTCAACTCCGACCCCTTCGAC
CCCGAGGTCATGGGCCCCGAGGTCCGCGACCGCCTCCTCAAGGGCGAGGACCTTCCCGTCACCA
CCACCAAGATCACCATGGTCGACCTGCCCCTCGGCGCCACCGAGGACAGGGTGTGCGGCACCAT

CGACATTGAGAAGGCGCTCACCGAAGGTGTCAAGGCGTTCGAGCCAGGCCTGCTCGCCAAGGCC
AACAGGGGGATACTGTATGTGGACGAGGTCAATCTGCTGGACGACCATCTGGTGGATGTTCTGC
TGGATTCCGCGGCTTCCGGGTGGAACACGGTGGAGAGGGAGGGCATCTCCATCTCCCACCCTGC
GCGGTTCATCCTCATTGGGTCCGGTAACCCGGAGGAAGGCGAGCTCCGGCCGCAGCTGCTGGAC
CGGTTCGGGATGCACGCGCAGGTCGGCACGGTCAGGGACGCGGAGCTGAGGGTGAAGATTGTG
GAGGAGAGGGCTCGGTTCGACAAGGACCCGAAAACGTTCCGGCAGTCCTACTTGGAGGAGCAA
GGGAAGCTCCAGGACCAGATCACATCCGCTCGGAGCAACCTCGGTTCTGTGCAGCTCGACCATG
ATCTCCGGGTTAAGATATCCCAGGTGTGTTCTGAGCTGAATGTGGATGGGCTGAGAGGAGACAT
TGTCACTAACAGGGCTGCCAAGGCGTTGGCTGCCCTAAAGGGAAGGGACATCGTGACAGTGGAG
GACATTGCCACCGTGATTCCCAACTGTTTGAGGCATCGGCTCCGTAAAGACCCACTCGAATCGAT
CGACTCGGGCTTGCTTGTAGTTGAGAAGTTTTATGAAGTCTTTGGCTAG

> TraesCS7B01G382800.3 (*TaCHLI-7B*)

ATGGCCATGGCCTCCCCGTTCTCCCCGGCCTCGGCCGCCGCCGCCTCGCCGGCCCTCTTCTCCGT
CTCCACCTCCCGCCCTCTCTCCCTCACCACCGCCGCGACCGCCGCCGTCTCAGCCCGGGCTCCGT
GCAGGGGCAGCAGAGGATTCCGCCGCGGCCGCTTCGCCGTCTGCAATGTCGCCGCCCCCTCCGC
CGCCGTGCAGGAGACCAAGCCGGCGGCGGCTGCGAAGGAGAGCCAGCGGCCGGTGTACCCGTT
CCCGGCGATCGTGGGGCAGGACGAGATGAAGCTCTGCCTGCTGCTCAACGTCATCGACCCCAAG
ATCGGCGGCGTCATGATCATGGGCGACCGGGGCACCGGCAAGTCCACCACCGTCCGCTCCCTCG
TCGACCTGCTCCCGGACATCAGCGTCGTTGTCGGCGACCCGTTCAACTCCGACCCCTACGACCCC
GAGGTCATGGGCCCCGAGGTCCGCGACCGCCTCCTCAAGGGCGAGGACCTTACCGTCACCACCA
CCAAGATCACCATGGTCGACCTGCCCCTCGGCGCCACCGAGGACAGGGTGTGCGGCACCATCGA
CATTGAGAAGGCGCTCACCGAAGGTGTCAAGGCGTTCGAGCCAGGCCTGCTTGCCAAGGCCAAC
AGGGGGATACTGTATGTGGACGAGGTTAATCTGCTGGACGACCATCTGGTGGATGTTCTGCTGG
ATTCCGCGGCTTCCGGGTGGAACACGGTGGAGAGGGAGGGCATCTCCATCTCCCACCCTGCGCG
GTTCATCCTCATTGGGTCCGGTAACCCGGAGGAAGGCGAGCTCCGGCCACAGCTGCTGGACCGG
TTCGGGATGCACGCGCAGGTCGGCACGGTCAGGGATGCGGAGCTGAGGGTGAAGATTGTAGAG
GAGAGGGCTCGGTTCGACAAGGACCCGAAAACGTTCCGGCAGTCCTACTTGGAGGAGCAAGGG
AAGCTCCAGGATCAGATCACATCGGCTCGGAGCAACCTCGGTTCTGTGCAGCTCGACCATGATC
TCCGGGTTAAGATATCCCAGGTGTGTTCTGAGCTGAATGTGGATGGGCTGAGAGGAGACATTGT
CACTAACAGGGCTGCCAAGGCGTTGGCTGCCTTGAAAGGAAGGGACATTGTGACAGTGGAGGA
CATTGCCACTGTGATCCCCAACTGTTTGAGGCATCGGCTCCGTAAAGACCCGCTCGAATCGATCG
ACTCGGGCTTGCTTGTAGTTGAGAAGTTTTATGAAGTCTTTGGCTAG

> TraesCS7D01G467500.1 (*TaCHLI-7D*)

ATGGCCATGGCCTCCCCGTTCTCCCCGGCGTCGGCCGCCGCCACCTCGCCGGCCCTCTTCTCCGT
CTCCACCTCCCGCCCACTCTCCCTCACCACCGCCGCGACCGCCGCCATCTCAGCCCGAGCTCCGT
CCAGGACCAGCAGAGGGTTCCGCCGCGGCCGCTTCGCCGTCTGCAATGTCGCCGCCCCCTCCGC
CGCCGTGCAGGAGACCAAGCCGGCGGCGGGCGCGAAGGAGAGCCAGCGGCCGGTGTACCCGTT
CCCGGCGATCGTGGGGCAGGACGAGATGAAGCTCTGCCTGCTGCTCAACGTCATCGACCCCAAG
ATCGGCGGCGTCATGATCATGGGCGACCGGGGCACCGGCAAGTCCACCACCGTCCGCTCCCTCG
TCGACCTGCTCCCGGACATCAGCGTCGTTGTCGGCGACCCGTTCAACTCCGACCCCTTCGACCCC
GAGGTCATGGGCCCCGAGGTCCGTGACCGCCTCCTCAAGGGCGAGGACCTTCCCGTGACCACCA
CCAAGATCACCATGGTCGACCTGCCCCTCGGCGCCACCGAGGACAGGGTGTGCGGCACCATCGA
CATTGAGAAGGCGCTCACCGAAGGTGTCAAGGCGTTCGAGCCAGGCCTGCTTGCCAAGGCCAAC
AGGGGGATACTGTATGTGGACGAGGTCAATCTGCTGGATGACCATCTGGTGGATGTTCTGCTGG
ATTCCGCGGCGTCTGGGTGGAACACGGTGGAGAGGGAGGGCATCTCCATCTCCCACCCTGCACG
GTTCATCCTCATTGGGTCCGGTAACCCGGAGGAAGGCGAGCTCCGGCCGCAGCTGCTGGACCGG

TTCGGGATGCACGCGCAGGTTGGCACGGTCAGGGACGCGGAGCTGAGGGTGAAGATTGTGGAG
GAGAGGGCTCGGTTCGACAAGGACCCAAAAACGTTCCGGCAGTCCTACTTGGAGGAGCAAGGG
AAGCTCCAGGACCAGATCACATCCGCTCGGAGCAACCTGGGCTCTGTGCAGCTCGACCATGATC
TCCGGGTTAAGATATCCCAGGTGTGTTCCGAGCTGAATGTGGATGGACTGAGAGGAGACATTGT
CACTAACAGGGCGGCCAAGGCGTTGGCTGCCTTGAAAGGAAGGGACATTGTGACAGTCGAGGA
CATTGCCACTGTGATCCCCAACTGTTTGAGGCATCGGCTCCGTAAAGACCCACTCGAATCGATCG
ACTCGGGCTTGCTTGTAGTTGAGAAGTTTATGAAGTCTTTGGCTAG

Figure S6. cDNA sequence of TraesCS7A02G480700.1 (*TaCHLI-7A*), TraesCS7B01G382800.3 (TaCHLI-7B) and TraesCS7D01G467500.1 (*TaCHLI-7D*).

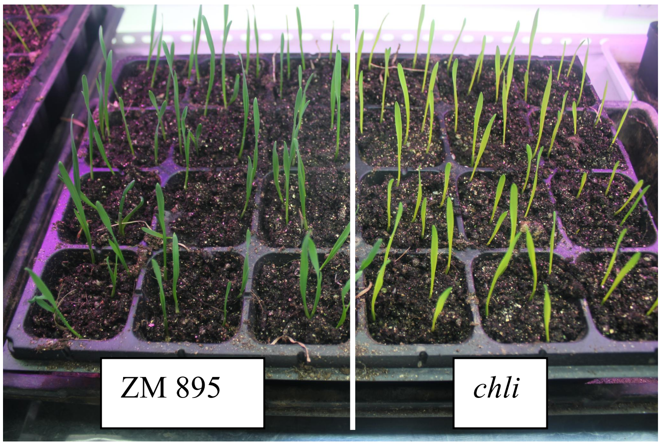

Figure S7. The phenotype of ZM 895 and *chli* mutant at seedling stage.

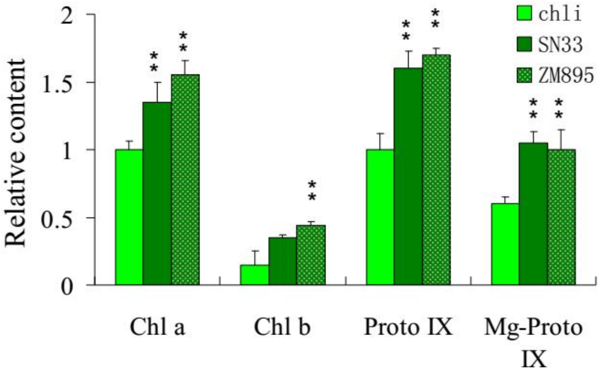

Figure S8. The content of Chl and Proto of *chli*, SN33 and ZM895 at one-leaf stage. ** indicate significant differences at $p < 0.01$.

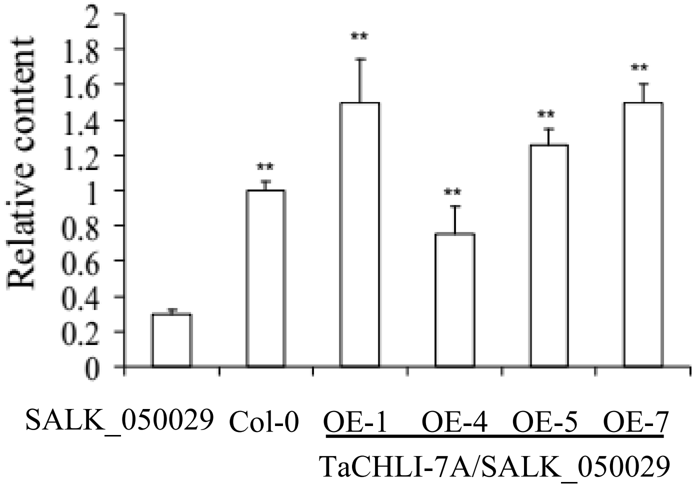

Figure S9. The content of Chl of homozygous T_2 generation transgenic plants. SALK_050029: CHLI mutant Arabidopsis; WT: wild type Arabidopsis; OE is overexpression. ** indicate significant differences at $p< 0.01$.

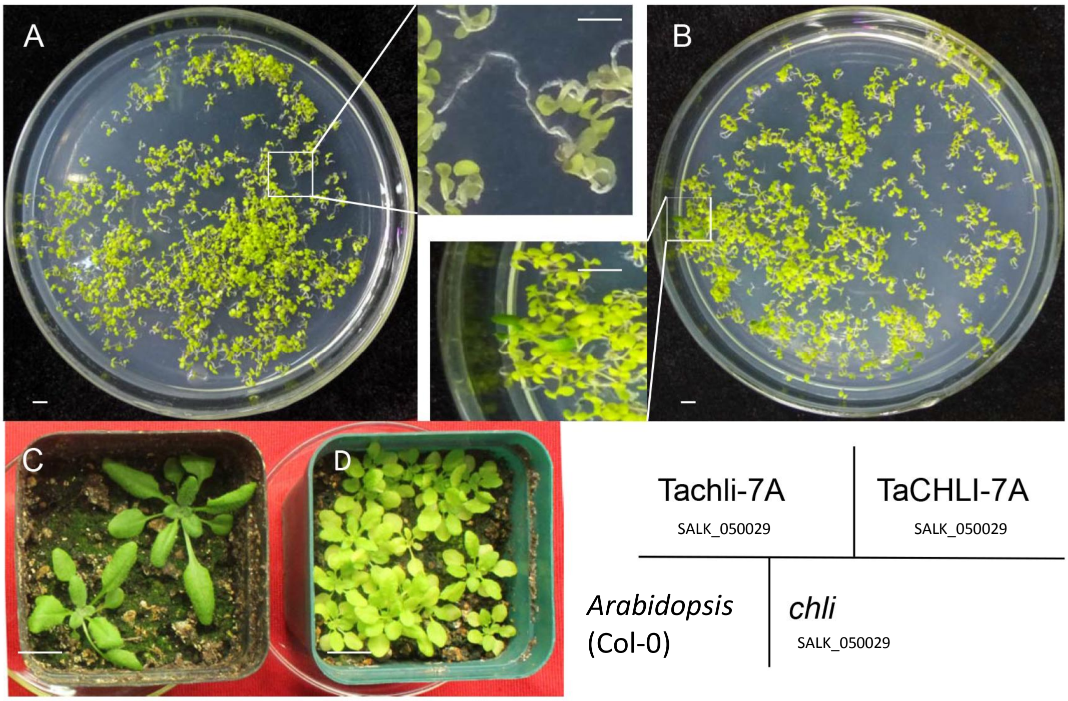

Figure S10. Phenotypes of *Arabidopsis* SALK_050029 mutant carrying *TaCHLI-7A* and *Tachli-7A* genes. **(A)** Transgenic SALK_050029 mutants carrying 35s*::Tachli-7A*. **(B)** Transgenic SALK_050029 mutants carrying 35s*::TCHLI-7A*. **(C)** 3-week old *Arabidopsis* (Col-0). **(D)** 3-week old SALK_050029 mutants Arabidopsis. Bars = 5 mm.

1. The method used in q-PCR:

Run the qPCR according to the following cycle procotol:
Enzyme activation 95°C, hold 3 min;

Denaturation 95°C 5 s
Annealing 60°C 20 s
} 40 cycles;

2. SNP filtering method:

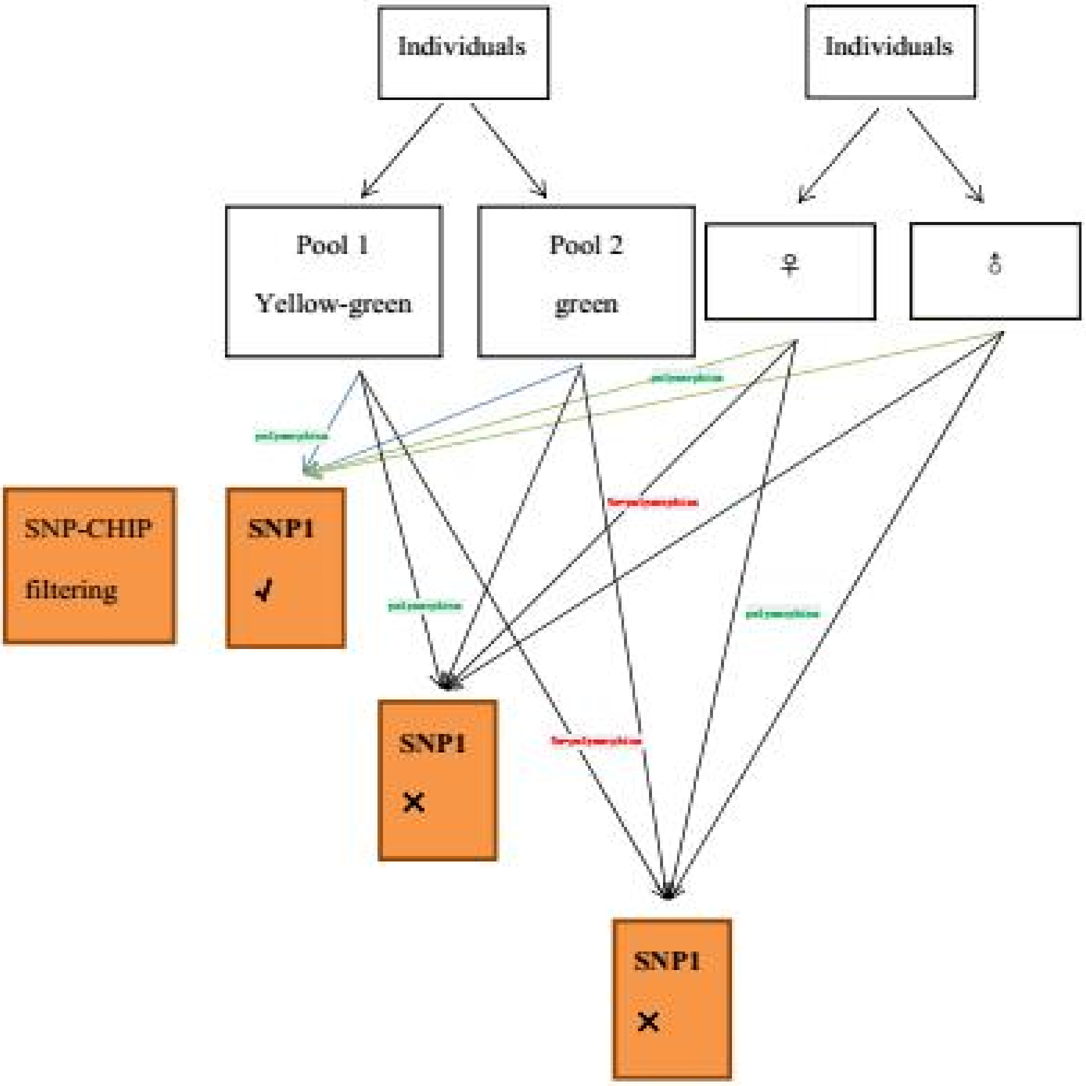

SNP filtration by using following method: i. deleting the SNP which were not detected in Chinese Spring wheat physical map; ii. deleting the SNP which were missing in one of two DNA pools; iii. Deleting the SNP which were no polymorphism between parental DNA or two DNA pool. The SNP distribution frequency ratio (polymorphism SNP numbers/total SNP mapped to the wheat physical map) on wheat chromosome physical map was calculated per 10 Mb physical intervals.

3. The process of identifying candidate gene:

First we locked this mutant gene in a 10 Mb interval in chromosome, the mutant chli exhibited a chlorophyll deficiency phenotype.
Then, we blast the chlorophyll synthesis relative genes by using the genes from rice and Arabidopsis.
Finally, chlorophyll synthesis relative gene TaCHLI was found in this 10 Mb interval. Therefore, we consider it as the candidate gene and more confirmed experiments were done.
Chlorophyll synthesis relative genes in rice, *Arabidopsis* and commom wheat:

Gene/protein	gene	correspond to rice (NCBI)	correspond to *Arabidopsis* (NCBI)	correspond to Common wheat (IWGSC RefSeq v1.0)	chromosome
Glutamyl tRNA reductase	*HEMA1*<br>*HEMA2*<br>*HEMA3*	 <br>Loc_Os10g35840	At1g58290<br>At1g09940<br>At2g31250	TraesCS1D01G165600<br>TraesCS1A01G173100<br>TraesCS1B01G058300	1A, 1B, 1D
Glutamate-1-semialdehyde aminotranase 2,1Aminomutase	*GSA1*<br>*GSA2*	 Loc_Os08g41990	At5g63570<br>At3g48730	TraesCS7A01G260800<br>TraesCS7B01G158800<br>TraesCS7D01G261800	7As, 7Bs, 7Ds
5-Aminolevlinate dehydratase	*HEMB1*<br>*HEMB2*		At1g69740<br>At1g44318	TraesCS6A01G107900<br>TraesCS6B01G136500<br>TraesCS6D01G095800	6As, 6Bs, 6Ds
Porphobilinogen deaminase	*HEMC*	Loc_Os02g07230	At5g08280	TraesCS6A01G143300<br>TraesCS6B01G171600<br>TraesCS6D01G132500	6As, 6Bs, 6Ds
Uroporphyrinogen III synthase	HEMD	Loc_Os03g08730	At2g26540	TraesCS4A01G002800<br>TraesCS4B01G302400<br>TraesCS4D01G300900	4Bl, 4Dl，4As
UroporphyrinogenIII decarboxylase	HEME1	Loc_Os01g43390	At3g14930	TraesCS3A01G224800<br>TraesCS3B01G254400<br>TraesCS3D01G228800	3Al, 3Bl, 3Dl
	HEME2	Loc_Os03g21990	At2g40490	TraesCS3A01G175600<br>TraesCS3B01G205300<br>TraesCS3D01G181100	3As, 3Bs, 3Ds
Coproporphyrinogen oxidase	HEMF1<br>HEMF2	Loc_Os04g52130	At1g03475<br>At4g03205	TraesCS2A01G426900<br>TraesCS2B01G447300<br>TraesCS2D01G425000	2Al,2Bl, 2Dl
PPO, protoporphyrinogen IX oxidase	HEMG1	Loc_Os01g18320	At4g01690	TraesCS5A01G045700<br>TraesCS5B01G049800<br>TraesCS5D01G055700	5As,5Bs,5Ds
	HEMG2	Loc_Os04g41260	At5g14220	TraesCS2A01G347900<br>TraesCS2B01G366300<br>TraesCS2D01G346200	2Al,2Bl, 2Dl
Mg-chelatase I subunit	ChL11<br>ChL12	Loc_Os03g36540	At4g18480<br>At5g45930	TraesCS7A01G480700<br>TraesCS7B01G382800<br>TraesCS7D01G467500	7Al, 7Bl, 7Dl
Mg-chelatase D subunit	CHLD	Loc_Os03g59640	At1g08520	TraesCS5A01G466100<br>TraesCS5B01G477800<br>TraesCS5D01G478900	5AL, 5Bl,5Dl
Mg-chelatase H subunit	CHLH	Loc_Os03g20700	At5g13630	TraesCS2A01G134000<br>TraesCS2B01G157600<br>TraesCS2D01G136200	2As,2Bs, 2Ds
Mg-protoporphyrin IX methyltransferase	ChLM	Loc_Os06g04150	At4g25080	TraesCS7A01G068500<br>TraesCS4A01G420600<br>TraesCS7D01G062900	7As, 4A, 7Ds
Mg-protoporphyrin IX monomethylaste cyclase	CHL27	Loc_Os01g17170	At3g56940	TraesCS3A01G191700<br>TraesCS3B01G219700<br>TraesCS3D01G194300	3As, 3Bs, 3Ds
8-vinyl reductase	DVR	Loc_Os03g22780	At5g18660	TraesCS6A01G403800<br>TraesCS6B01G447700<br>TraesCS6D01G387300	6AL, 6Bl,6Dl
NADPH-protochlorophyllide oxidoreductase	PORA	Loc_Os04g58200	At5g54190	TraesCS2A01G590600<br>TraesCS2B01G593000<br>TraesCS2D01G563600	2A, 2B, 2D

PORB		Loc_Os10g35370	At4g27440	TraesCS1A01G171000 TraesCS1B01G186300 TraesCS1D01G168700	1Al, 1Bl, 1Dl
PORC		Loc_Os10g35730	At1g03630	TraesCS1A01G171000 TraesCS1B01G186300 TraesCS1D01G168700	1Al, 1Bl, 1Dl
Chlorophyll synthase	ChLG	Loc_Os05g28200	At3g51820	TraesCS1A01G224700 TraesCS1B01G237700 TraesCS1D01G226100	1Al, 1Bl, 1Dl
Chlorophyll a oxygenase	CAO	Loc_Os10g41780	At1g44446	TraesCS3A01G506200 TraesCS3B01G574300 TraesCS3D01G514100	3Al, 3Bl, 3Dl

* correspond to Common wheat gene based on IWGSC RefSeq v1.0.

4. The measurement of proto ix and mg-proto ix by HPLC

Table1. Analyte: proto IX

Sample Name	Sample Type	Area (Y*sec)	(min)	Target [Conc]. (pmol/mL)	Calculated Conc.samp (pmol/mL)
WCSWCJ20191212-S	Standard	1453	25.8	50	
WCSWCJ20191212-1	M1-1	5017	25.4	N/A	173
WCSWCJ20191212-2	M1-2	4742	25.5	N/A	163
WCSWCJ20191212-3	M1-3	5510	25.5	N/A	190
WCSWCJ20191212-4	M2-1	6699	25.7	N/A	231
WCSWCJ20191212-5	M2-2	7453	25.4	N/A	257
WCSWCJ20191212-6	M2-3	7801	25.9	N/A	269
WCSWCJ20191212-7	S1-1	11107	25.2	N/A	383
WCSWCJ20191212-8	S1-2	10701	25.4	N/A	369
WCSWCJ20191212-9	S1-3	11310	25.9	N/A	390
WCSWCJ20191212-10	S2-1	10469	25.6	N/A	361
WCSWCJ20191212-11	S2-2	10092	25.3	N/A	348
WCSWCJ20191212-12	S2-3	10730	25.7	N/A	370
WCSWCJ20191212-13	S6-1	38222	25.2	N/A	1318
WCSWCJ20191212-14	S6-2	42050	25.9	N/A	1450
WCSWCJ20191212-15	S6-3	37091	25.3	N/A	1279
WCSWCJ20191212-16	S7-1	36192	25.4	N/A	1248
WCSWCJ20191212-17	S7-2	34655	25.6	N/A	1195
WCSWCJ20191212-18	S7-3	37265	25.2	N/A	1285
WCSWCJ20191212-19	S8-1	39411	25.9	N/A	1359
WCSWCJ20191212-20	S8-2	37294	25.7	N/A	1286
WCSWCJ20191212-21	S8-3	39730	25.8	N/A	1370
WCSWCJ20191212-22	S9-1	35351	25.4	N/A	1219
WCSWCJ20191212-23	S9-2	37700	25.0	N/A	1300
WCSWCJ20191212-24	S9-3	42224	25.7	N/A	1456
WCSWCJ20191212-25	S11-1	44660	26.0	N/A	1540
WCSWCJ20191212-26	S11-2				
WCSWCJ20191212-27	S11-3	38541	25.4	N/A	1329
WCSWCJ20191212-28	S13-1				
WCSWCJ20191212-29	S13-2	44689	25.7	N/A	1541
WCSWCJ20191212-30	S13-3	42311	25.9	N/A	1459
WCSWCJ20191212-31	S15-1	44892	25.5	N/A	1548
WCSWCJ20191212-32	S15-2	39875	25.4	N/A	1375
WCSWCJ20191212-33	S15-3	40687	26.2	N/A	1403
WCSWCJ20191212-34	S19-1	39382	25.4	N/A	1358

Sample Name	Sample Type	Area (Y*sec)	(min)	Target [Conc]. (pmol/mL)	Calculated Conc.samp (pmol/mL)
WCSWCJ20191212-35	S19-2	41383	25.2	N/A	1427
WCSWCJ20191212-36	S19-3	44515	25.3	N/A	1535

Standard: 50 pmol/mL
E400/F630
M1: mutant chli one-leaf stage
M2: mutant chli three-leaf stage
S1: SN33 one-leaf stage
S2: SN33 three-leaf stage
S6-S19: 6h-19h
Area (Y*sec): Y= Corresponding luminous intensity in HPLC Y axis.

Table2. Analyte: Mg-proto IX

Sample Name	Sample Type	Area (Y*sec)	(min)	Target [Conc]. (pmol/mL)	Calculated Conc.samp (pmol/mL)
WCSWCJ20191212-S	Standard	7248	15.7	50	
WCSWCJ20191212-1	M1-1	16815	15.4	N/A	116
WCSWCJ20191212-2	M1-2	14931	15.6	N/A	103
WCSWCJ20191212-3	M1-3	13191	15.1	N/A	91
WCSWCJ20191212-4	M2-1	29427	15.7	N/A	203
WCSWCJ20191212-5	M2-2	34356	15.4	N/A	237
WCSWCJ20191212-6	M2-3	27397	15.8	N/A	189
WCSWCJ20191212-7	S1-1	45662	15.3	N/A	315
WCSWCJ20191212-8	S1-2	40734	15.7	N/A	281
WCSWCJ20191212-9	S1-3	49286	15.7	N/A	340
WCSWCJ20191212-10	S2-1	45083	15.7	N/A	311
WCSWCJ20191212-11	S2-2	50881	15.2	N/A	351
WCSWCJ20191212-12	S2-3	48852	15.2	N/A	337
WCSWCJ20191212-13	S6-1	59289	15.5	N/A	409
WCSWCJ20191212-14	S6-2	56100	15.7	N/A	387
WCSWCJ20191212-15	S6-3	62623	15.4	N/A	432
WCSWCJ20191212-16	S7-1	57404	15.9	N/A	396
WCSWCJ20191212-17	S7-2	65522	15.6	N/A	452
WCSWCJ20191212-18	S7-3	66827	15.4	N/A	461
WCSWCJ20191212-19	S8-1	65377	15.5	N/A	451
WCSWCJ20191212-20	S8-2	67406	15.6	N/A	465
WCSWCJ20191212-21	S8-3	62188	15.2	N/A	429
WCSWCJ20191212-22	S9-1	58999	15.2	N/A	407
WCSWCJ20191212-23	S9-2	64942	15.3	N/A	448
WCSWCJ20191212-24	S9-3	60303	15.4	N/A	416
WCSWCJ20191212-25	S11-1	62333	15.5	N/A	430
WCSWCJ20191212-26	S11-2				
WCSWCJ20191212-27	S11-3	61898	15.3	N/A	427
WCSWCJ20191212-28	S13-1				
WCSWCJ20191212-29	S13-2	63492	15.3	N/A	438
WCSWCJ20191212-30	S13-3	62188	15.8	N/A	429
WCSWCJ20191212-31	S15-1	66102	15.6	N/A	456
WCSWCJ20191212-32	S15-2	60158	15.4	N/A	415

Sample Name	Sample Type	Area (Y*sec)	(min)	Target [Conc]. (pmol/mL)	Calculated Conc.samp (pmol/mL)
WCSWCJ20191212-33	S15-3	56969	15.2	N/A	393
WCSWCJ20191212-34	S19-1	69726	15.5	N/A	481
WCSWCJ20191212-35	S19-2	65377	15.5	N/A	451
WCSWCJ20191212-36	S19-3	60738	15.8	N/A	419

Standard: 50 pmol/mL
E416/F595
M1: mutant chli one-leaf stage
M2: mutant chli three-leaf stage
S1: SN33 one-leaf stage
S2: SN33 three-leaf stage
S6-S19: 6h-19h
Area (Y*sec): Y= Corresponding luminous intensity in HPLC Y axis

The first peak in ~15 min is mg-proto ix, second peak in ~25min is proto ix

WCSWCJ20191212-S	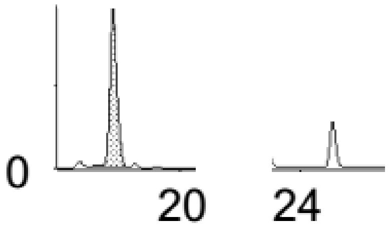
WCSWCJ20191212-1	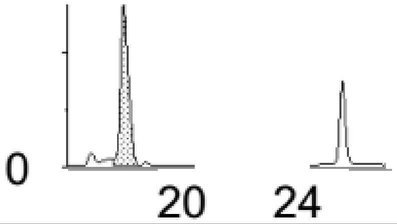
WCSWCJ20191212-2	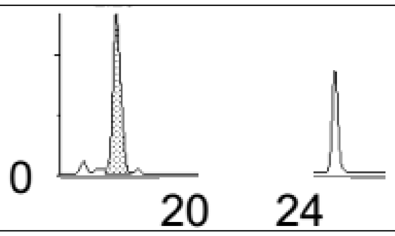
WCSWCJ20191212-3	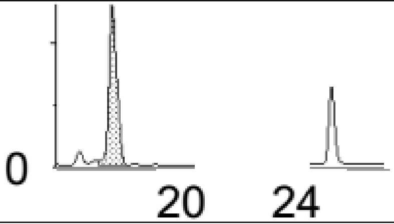

WCSWCJ20191212-4	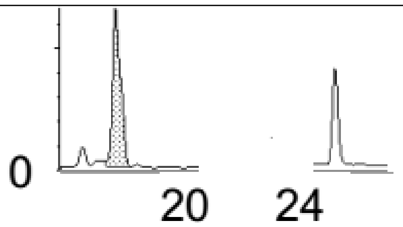
WCSWCJ20191212-5	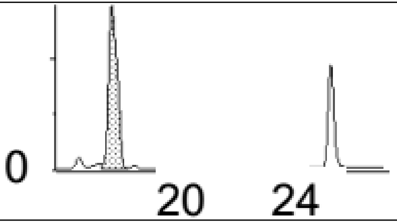
WCSWCJ20191212-6	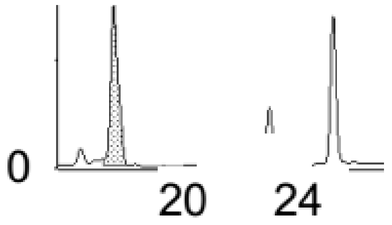
WCSWCJ20191212-7	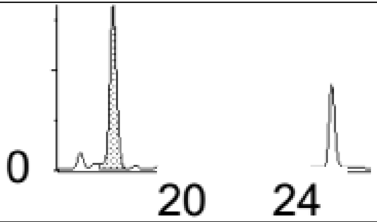
WCSWCJ20191212-8	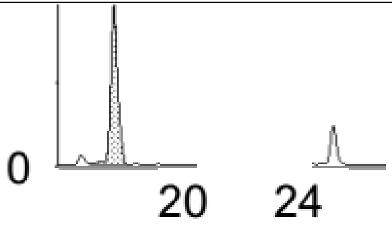
WCSWCJ20191212-9	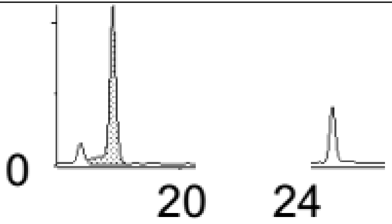
WCSWCJ20191212-10	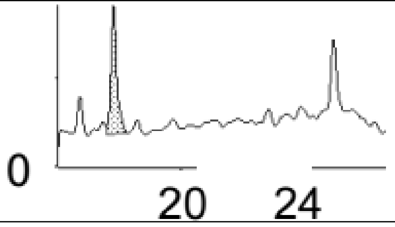

WCSWCJ20191212-11	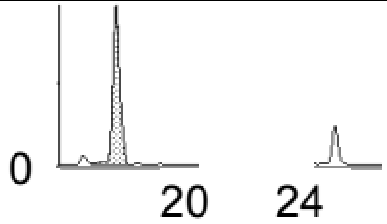
WCSWCJ20191212-12	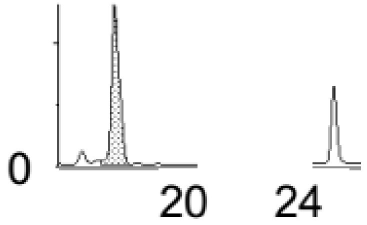
WCSWCJ20191212-13	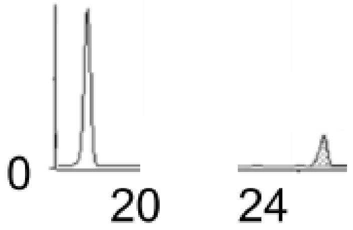
WCSWCJ20191212-14	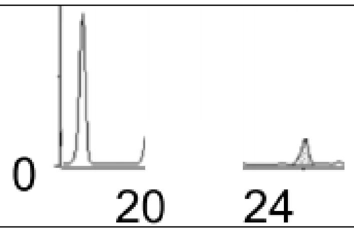
WCSWCJ20191212-15	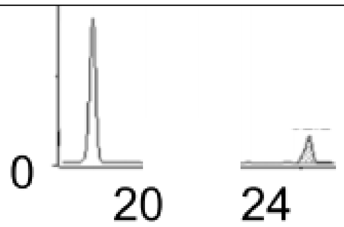
WCSWCJ20191212-16	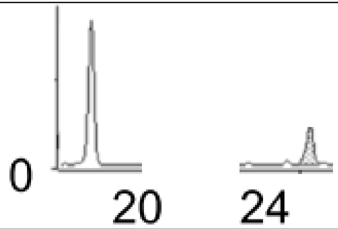
WCSWCJ20191212-17	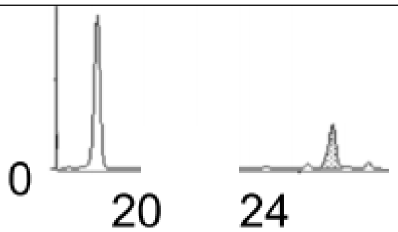

WCSWCJ20191212-18	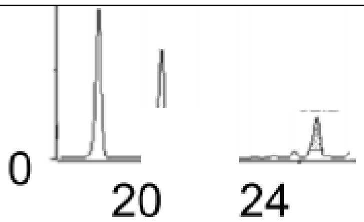
WCSWCJ20191212-19	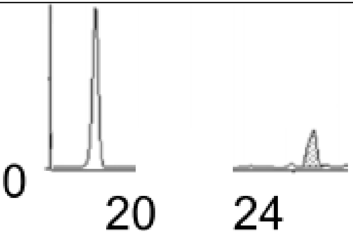
WCSWCJ20191212-20	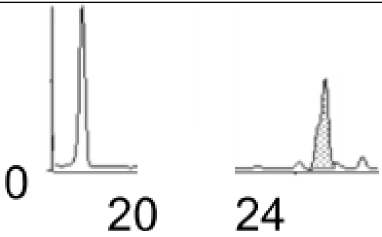
WCSWCJ20191212-21	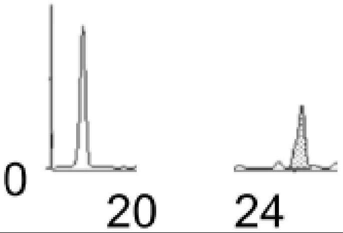
WCSWCJ20191212-22	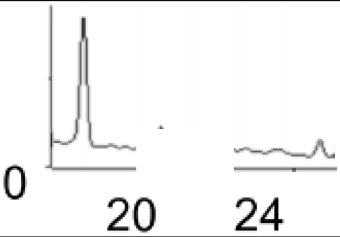
WCSWCJ20191212-23	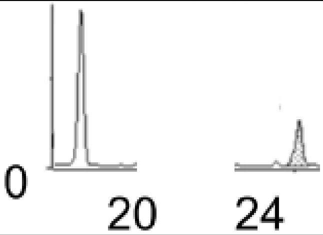

WCSWCJ20191212-24	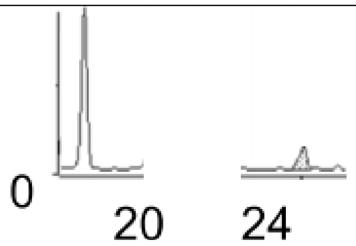
WCSWCJ20191212-25	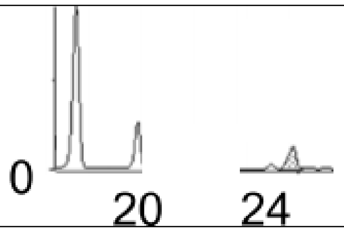
WCSWCJ20191212-26	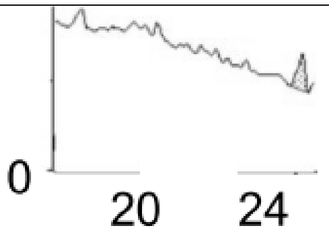
WCSWCJ20191212-27	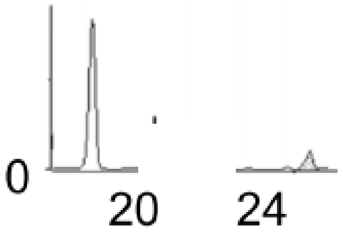
WCSWCJ20191212-28	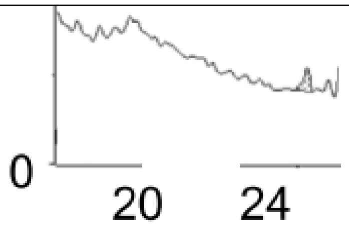
WCSWCJ20191212-29	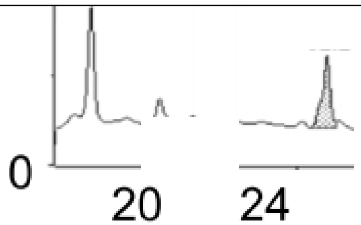
WCSWCJ20191212-30	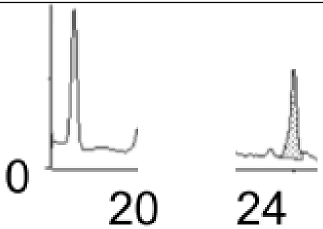

WCSWCJ20191212-31	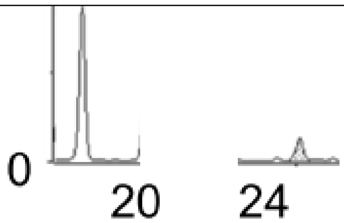
WCSWCJ20191212-32	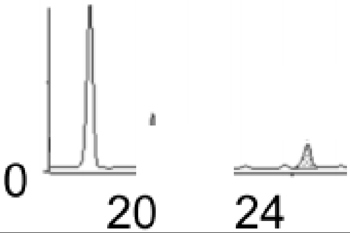
WCSWCJ20191212-33	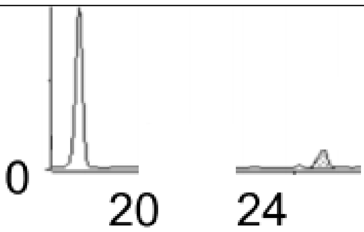
WCSWCJ20191212-34	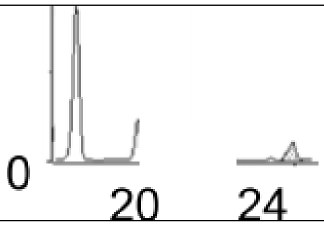
WCSWCJ20191212-35	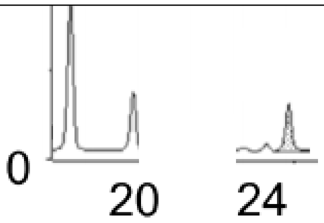
WCSWCJ20191212-36	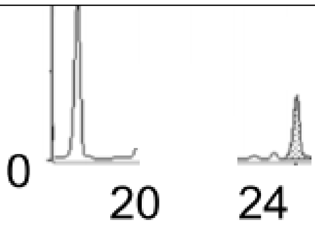
